# Modeling premature aging in yeast via the expression of Progerin

**DOI:** 10.18632/aging.206367

**Published:** 2026-04-03

**Authors:** Zachery R. Belak, Christopher Ptak, Rachel E. Harris, Rick Wozniak, Christopher H. Eskiw, Troy AA. Harkness

**Affiliations:** 1 Department of Food and Bioproduct Sciences, University of Saskatchewan, Saskatoon S7N 5C9, Saskatchewan, Canada; 2 Department of Biochemistry, Microbiology and Immunology, University of Saskatchewan, Saskatoon S7N 5E5, Saskatchewan, Canada; 3 Department of Cell Biology, University of Alberta, Edmonton T6G 2H7, Alberta, Canada; 4 Department of Anatomy and Cell Biology, University of Saskatchewan, Saskatoon S7N 5E5, Saskatchewan, Canada; 5 Department of Medicine, University of Alberta, Edmonton T6G 2B7, Alberta, Canada

**Keywords:** Hutchinson-Gilford Progeria Syndrome, yeast, Progerin, Lamin A, premature aging

## Abstract

The budding yeast, *Saccharomyces cerevisiae*, is an excellent model for studying mechanisms governing aging, with many genetic and biochemical pathways highly conserved. Here, we established a model of premature aging by expressing galactose inducible EGFP fusions of the cytotoxic protein Progerin, and its wild type form, Lamin A (LMNA), in yeast. Progerin is the driving force behind the incurable disease Hutchinson-Gilford Progreria Syndrome, which causes children to age 8 times faster than normal. We observed that EGFP-Progerin and EGFP-LMNA both localized to the yeast nuclear membrane. Progerin expression, but not LMNA, caused yeast cells to grow slowly, experience increased genome instability, and exhibit reduced chronological lifespan. The Lamin A protein had a half-life of 4 hours in yeast, whereas Progerin remained stable for 24 hours, with Progerin accumulating in mother cells, parallel to other long lived asymmetrically retained proteins (LARPS). The select segregation of LARPS into mother cells is associated with the aging program, as this potentially protects daughter cells, ensuring a pristine start and a full lifespan. Using flow cytometry with cells stained with Wheat Germ Agglutinin to score buds scars and relative yeast age, we confirmed that Progerin, but not LMNA, was retained specifically in aging mother cells. Taken together, expression of Progerin in yeast cells mimics what is observed in human cells, establishing yeast as a powerful model to discover genetic mechanisms driving premature and normal aging.

## INTRODUCTION

The brewing yeast, *Saccharomyces cerevisiae*, has been used in decades of research and has provided fundamental insights into basic cellular and molecular biology into aging and disease [1–4]. This seemingly simple single-cellular organism has many of the same molecular pathways governing lifespan as complex multicellular organisms, but in simpler forms [5–7]. Yeast lifespan can be measured using either a chronological lifespan (CLS) assay that measures the length of time a population of cells remains metabolically active, or by a replicative lifespan (RLS) assay, which measures how many daughter cells a single mother cell can produce [8–11]. In addition, yeast are easily manipulated genetically via the addition of exogenous genes or the deletion of endogenous genes [12, 13]. As such, yeast make an excellent model for not only studying the underlying mechanisms governing cellular aging, but also for screening new compounds or strategies with the potential to mitigate cellular aging.

One of the major causes of aging is the accumulation of proteins within the cell. Numerous normal or mutant proteins can cause this; however, the accumulation in the nucleus, in particular the nuclear periphery, is a site interest [14–20]. The accumulation of Progerin, the driver of Hutchinson Gilford Progeria Syndrome (HGPS) in humans, within the nuclear membrane, leads to massively accelerated cellular aging [21–23]. Progerin results from a mutation in the *Lamin A/C* (LMNA) gene, which activates a cryptic splice site in the transcript, generating an internal 50 amino acid deletion of the protein. This internal deletion prevents the cleavage and release of LMNA from the nuclear periphery, causing a thickening of the membrane and accumulation of Progerin. Progerin accumulation leads to several nuclear defects, including a loss of heterochromatin, decreased rates of DNA repair, global disruption of genome organization, and altered gene expression [24, 25]. Interestingly, disruption of cellular nutrient sensing pathways with compounds such as rapamycin (RAP) or a decrease in nutrient/energy availability, leads mammalian cells to degrade protein accumulations [19, 26], such as Progerin, indicating that targeting nutrient sensing may be key to stimulating cells to remove these cytotoxic proteins.

Although numerous essential cellular processes are conserved from yeast to humans, there are still significant mechanisms that differ. One of the most striking differences is mitosis, where the nuclear envelope is dissolved prior to chromosomal separation in mammalian cells, but maintained in budding yeast, resulting in a closed mitosis [27]. In addition, yeast do not have an identified lamin-like protein, or a lamina structure, within their nuclear envelopes [28]. This provides us with an opportunity to develop a novel yeast system as a model to study the impact of nuclear envelope protein accumulation on yeast lifespan using an inducible promoter system.

Given the impact of protein accumulation on cellular aging, in particular that of Progerin accumulation, the overarching aim of this research was to develop a rapid system to test compounds or strategies to reverse age-related cellular phenotypes of protein accumulation. To achieve this, we expressed enhanced green fluorescent protein (EGFP) tagged versions of wild type human LMNA and Progerin, under the control of a galactose inducible promoter, and measured the impact on population growth and cell survival. We determined that, although yeast do not express lamin proteins similar to those in human cells, both EGFP-LMNA and EGFP-Progerin localized to the nuclear envelope. However, both EGFP-LMNA and EGFP-Progerin were found to localize to discrete accumulations within the nuclear membrane. Cells expressing EGFP-Progerin, but not EGFP-LMNA, were slow growing and had a shortened chronological lifespan. Using a colony reversion assay, we observed that EGFP-Progerin, but not EGFP-LMNA, had a significantly higher impact on mutation rates. Removal of galactose had the predictable effect of decreased EGFP-tagged molecules within the cells; however, microscopic analysis indicated that cells that retained these proteins had comparable levels to those in induced cultures. Furthermore, cells that maintained high levels of EGFP-Progerin were associated with higher numbers of bud scar, but not EGFP-LMNA or EGFP alone. Our observations support that yeast expressing EGFP-Progerin serves as an excellent model for testing new strategies for stimulating the removal of cytotoxic protein accumulations that cause premature cellular aging.

## RESULTS

### Lamin A and Progerin localize to the nuclear membrane, with Progerin slowing growth, reducing chronological lifespan, and increasing genomic instability in yeast

To determine if EGFP-LMNA and EGFP-Progerin localize to the nuclear membrane of yeast cells (strain BY4741) parallel to that observed in human cells, we expressed N-terminal EGFP-tagged human Lamin A (LMNA), or the minigene product Progerin. This minigene lacks an internal 300 nucleotide sequence, mimicking the point mutation at position 606 in human cells that activate a cryptic splice site, resulting in the deletion of the same 50 amino acids. Transgenes were expressed under the control of the *GAL1* promoter using optimized media containing 2% w/v raffinose and 0.2% galactose for 4 h at 30° C. Raffinose can be broken down to sucrose and galactose [29], providing a consistent amount of galactose that can be delivered to a cell. We used 0.2% galactose to minimize the expression of LMNA and Progerin in order to limit any off effects of protein overexpression. Following total protein extraction, protein equivalents were loaded onto either 8% gels for EGFP expressing cells, or 12% gels for EGFP-LA or EGFP-Progerin expressing cells (Figure 1A, lower panel). Three transformants were selected from each transformation plate, subjected to the above expression conditions, with extracted proteins analyzed by Western blotting with antibodies against EGFP. These data demonstrate that the cells consistently expressed abundant amounts of recombinant protein of the expected sizes based on sequence analysis (Figure 1A, upper panels; see Supplementary Figure 3 for an image of an uncropped blot). Analysis of the western blots performed with 3 biological repeats shows that expression of EGFP-LMNA and EGFP-Progerin was robust within the population (Figure 1B). Quantification of the amount of protein within the population, normalized to total protein loading, indicates that there may be a trend towards more EGFP-Progerin within this population of cells. This indicates two possibilities: i) more GFP-Progerin is expressed or; ii) EGFP-Progerin is more stable than EGFP-LA. Statistical analysis demonstrates that the differences in protein content is not within a confidence interval of 95% (p-value > 0.05). Given that the genes are under the control of the same promoter and that the assays were performed in triplicate, this would indicate that if any significant amounts of EGFP-Progerin were in higher amounts within the population, this would be the result of stability and no other factors such as increased transcription from one vector vs. the others.

**Figure 1:**
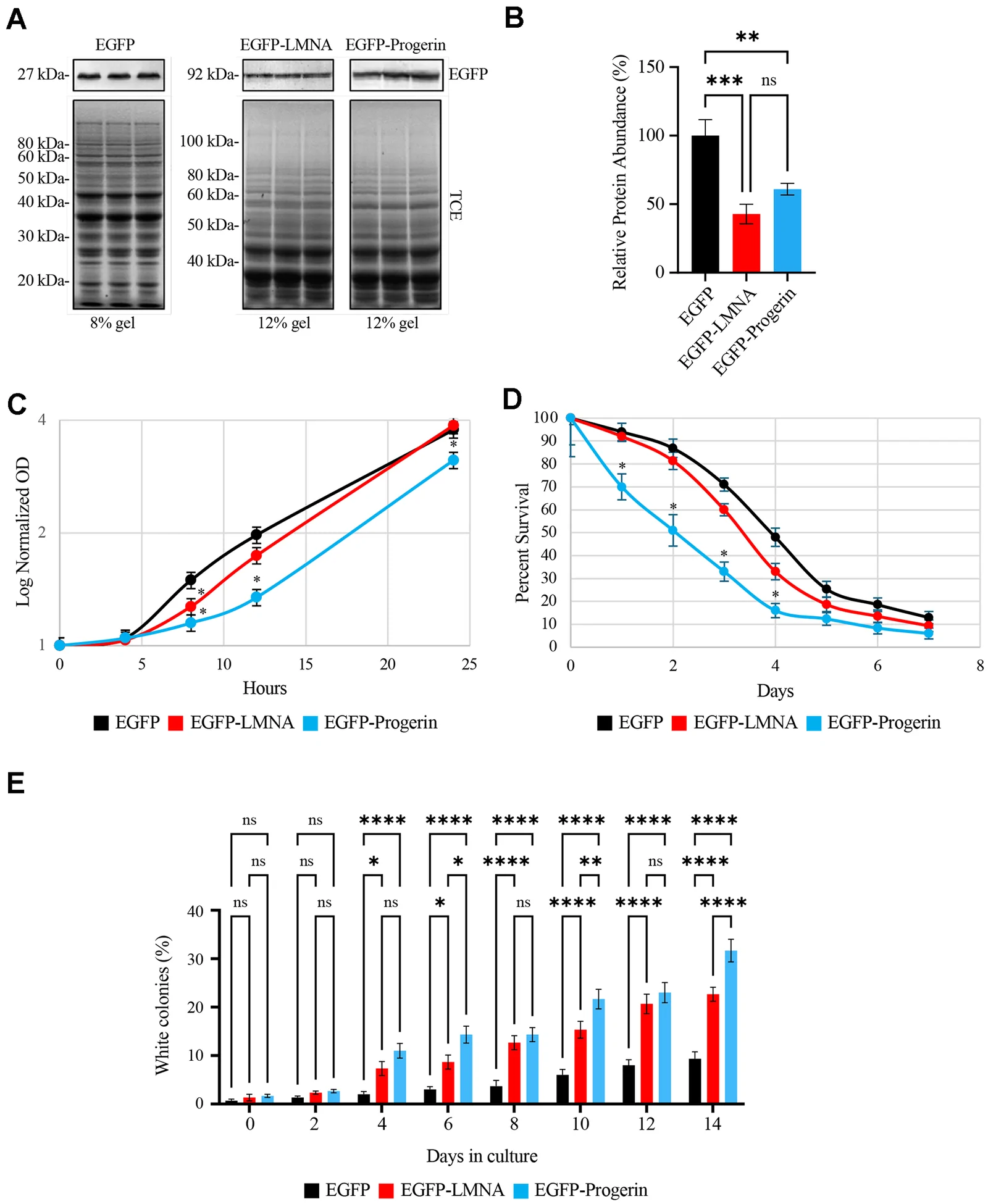
**Ectopically expressed EGFP-LMNA and EGFP-Progerin are targeted to the nuclear membranes of yeast cells with EGFP-Progerin disrupting cell growth, decreasing chronological lifespan and increasing genome instability.** pYES vectors containing galactose inducible promoters and encoding either EGFP, EGFP-LMNA or EGFP-Progerin were transformed into BY4741 yeast and expression induced in galactose containing media. (**A**) 3 colony transformants were selected from plates and grown in liquid media overnight, with proteins isolated for Western blotting for EGFP. Total protein (top panels - stained with trichloroethanol (TCE)) was used for loading controls, followed by Western blotting for EGFP (bottom panel – EGFP antibody). EGFP proteins were separated on 8% SDS-PAGE gels, while proteins for EGFP-LMNA and EGFP-Progerin are separated on 12% gels. The EGFP-LMNA and EGFP-Progerin lanes were cropped from the same gel. (**B**) The triplicate western bands for EGFP-LMNA and EGFP-Progerin were scanned and quantified. Values are presented as mean ± SD for n=3 and were analyzed with an ordinary one-way ANOVA with *post hoc* Tukey's multiple comparisons test where p-value < 0.01**, p-value < 0.001***, and p-value < 0.0001****. (**C**) Cultures of yeast expressing EGFP (black), EGFP-LMNA (red) or EGFP-Progerin (blue) were grown in 2% Raffinose/0.2% Galactose supplemented liquid media (n = 3) with OD_600_ readings collected at the times shown. Log normalized OD readings were plotted for the time points. Error bars represent standard error of the mean. * represents p-values < 0.05 via 2 tailed t-test. The OD_600_ at 4, 8, 12 and 24 hours were plotted for growth curves conducted in 2% RAF/0.2% GAL, as shown in Supplementary Figure 1. (**D**) Chronological lifespan assays were conducted using methylene blue on yeast expressing EGFP (black), EGFP-LMNA (red) and EGFP-Progerin (blue) and the percent cell survival over 7 days plotted. n=3. * represents 2-tailed t-test p-value < 0.05. Comparable CLS experiments performed using the standard colony counting method are shown in Supplementary Figure 2 (n=3). (**E**) Colony reversion assays were performed by expressing EGFP, EGFP-LMNA or EGFP-Progerin into the YPH500 strain carrying a point mutation in the *ADE2* gene. Westerns confirming correct expression of the proteins is shown in Supplementary Figure 3. This leads to yeast producing red coloration on YPD media. Red and white colonies were counted and the percentage of white colonies from EGFP (black), EGFP-LMNA (red) and EGFP-Progerin (blue) yeast was determined. The percentage of white colonies (y-axis) were scored over each day (x-axis) of the assay. Error bars - standard error of the mean (n=3). * represents p-value < 0.05, and ** via 2 tailed t-test.

Progerin is known to impair cell growth when expressed in human cells [30, 31]. To test whether Progerin impacts yeast cell growth, growth rates of EGFP-LMNA and EGFP-Progerin expressing cells were analyzed by monitoring the optical density of triplicate cultures growing in 2% RAF/0.2% GAL at 30° C and compared to cultures expressing EGFP alone (Figure 1C). EGFP alone was used as a base line control to eliminate any effects of EGFP. Overall, cells grown in 2% RAF/0.2% GAL grew significantly slower than cells grown in media containing glucose, regardless of which gene was expressed (data not shown), as raffinose is a complex sugar that is not as readily utilized as the simple sugar glucose [32]. EFGP-LMNA and EFGP-Progerin were not expressed in glucose supplemented media and did not influence the growth of cells maintained in 2% glucose (data not shown). Cells in 2% RAF/0.2% GAL entered log phase after approximately 4 h in culture and had not entered stationary phase within the 24h timecourse (Figure 1C). At 4 h after inoculation into 2% RAF/0.2% GAL media, both EGFP-LMNA and EGFP-Progerin expressing cells showed no significant decrease in growth relative to EGFP expressing control cells (Figure 1C and Supplementary Figure 1). After 8 h in culture, there was a significant difference in growth rate between LMNA and Progerin expressing cells compared to EGFP-expressing control cells. However, at 12 and 24 hours, there was a significant decrease in the density of cultures expressing EGFP-Progerin relative to EGFP-LMNA and EGFP alone. Although it is difficult to make a direct comparison, our data indicates that yeast growth is impacted by Progerin parallel to that observed previously in mammalian cells.

We next used chronological lifespan (CLS) assays to determine whether LMNA or Progerin impacted yeast lifespan (Figure 1D). CLS measures how long a population of stationary phase yeast cells can remain metabolically active [33]. Overall, there was a trend towards reduction in lifespan of LMNA expressing cells compared to control cells, but it did not reach a confidence interval of < 0.05. EGFP-Progerin conferred a much greater decline in CLS (p-value < 0.05), using the methylene blue CLS method (Figure 1D and Supplementary Figure 2 shows results using the standard colony forming unit method to measure CLS). We performed the experiment using minimal media (MSM) rather than RSM in order to reduce the length of time cells remain metabolically active in stationary phase, as previously described [34]. The number of viable cells present in cultures expressing EGFP-Progerin was significantly reduced relative to both control and EGFP-LMNA expressing cells at days 1 – 4, while by day 5 viable cell numbers in all cultures had fallen to a low level which was no longer statistically significant. EGFP-Progerin expressing cultures were reduced to 50% viability after 2 days while control cultures decreased to 50% viability after approximately 4 days, indicating a 2-fold decrease in their CLS.

Genomic instability is another known consequence of Progerin expression in human cells [34]. To test if this was also the case in yeast, we analyzed the impact of EGFP-LMNA and EGFP-Progerin on genomic instability in yeast using a colony reversion assay [35, 36]. This assay relies on measuring the rate of spontaneous reversion of an inactivating point mutation in the *ADE2* gene. Strains such as YPH500, which contain the *ade2-101_ochre* point mutation, are adenine auxotrophs that produce a truncated non-functional Ade2 protein. Without functional Ade2, an intense red colored substrate accumulates and confers a red color to the colonies of *ade2*-*101_ochre* strains. The *ade2-101_ochre* point mutation, specifically, is readily prone to spontaneous reversion, restoring wild type Ade2 function, leading to production of white colonies. However, mutations that impact upstream and intragenic sequences, as well as loss of mitochondrial DNA, mutations that increase oxidative stress, or mutations or growth conditions that lead to V-ATPase disassembly (such as on raffinose), could also lead to spontaneous reversion of red to white colony color. While we used raffinose media to induce the EGFP fusion proteins, the red/white conversion was much greater in cells expressing LMNA and Progerin. Red vs. white colonies can be easily identified and counted on agar media, and the frequency of this reversion under various conditions has become accepted as a measure of genomic stability [35].

To measure the effect of Progerin expression on genomic stability, we transformed the EGFP, EGFP-LMNA, and EGFP-Progerin expressing vectors into YPH500. We first verified the expression of EGFP-LMNA and EGFP-Progerin in YPH500 in 2% RAF/0.2% GAL using Western blot analysis (Supplementary Figure 3). These data show that all three proteins are expressed in this strain under these conditions. We then proceeded to use this strain in red/white colony assays to probe genomic stability. After 4 days in culture there was a significant and substantial increase in the number of white colonies exhibiting the *ade2-101_ochre* reversion in cultures expressing EGFP-LMNA or EGFP-Progerin compared to control cells, indicating increased genomic instability. Overall, the frequency of reversion was higher in cultures expressing EGFP-Progerin than Lamin A (Figure 1E). We observed a statistically significant increase in reversion rate in EGFP-Progerin expressing cultures relative to EGFP-LMNA at 6, 10, and 14 days in culture, but not at 4, 8 or 12 days. Although increased colony reversion was observed in GFP-LMNA expression cells compared to empty vector control, colony reversion trended higher at all time points in EGFP-Progerin expressing cells. This is consistent with the results observed above where LMNA displays a trend demonstrating a decrease in growth and lifespan compared to controls that did not reach statistical significance. Although LMNA does have limited impacts on these yeast phenotypes, GFP-Progerin had much greater impacts.

To analyze the localization of LMNA and Progerin expressed in yeast, aliquots of cultures were removed, fixed, and subjected to fluorescence microscopy (Figure 2A). The DNA-binding dye Hoechst 33342 (H33342) was used to visualize nuclear DNA (blue). To mark bud scars on the cell periphery, cells were further stained with wheat germ agglutinin (WGA) conjugated to Alexa 555 dye. EGFP was visualized using its endogenous fluorescence. Cells expressing the EGFP control showed a diffuse signal distributed evenly throughout the cytoplasm (Figure 2A, left panel). Cells expressing EGFP-LMNA and EGFP-Progerin showed EGFP signal localized to the nuclear periphery (Figure 2A, the middle and right panels).

**Figure 2:**
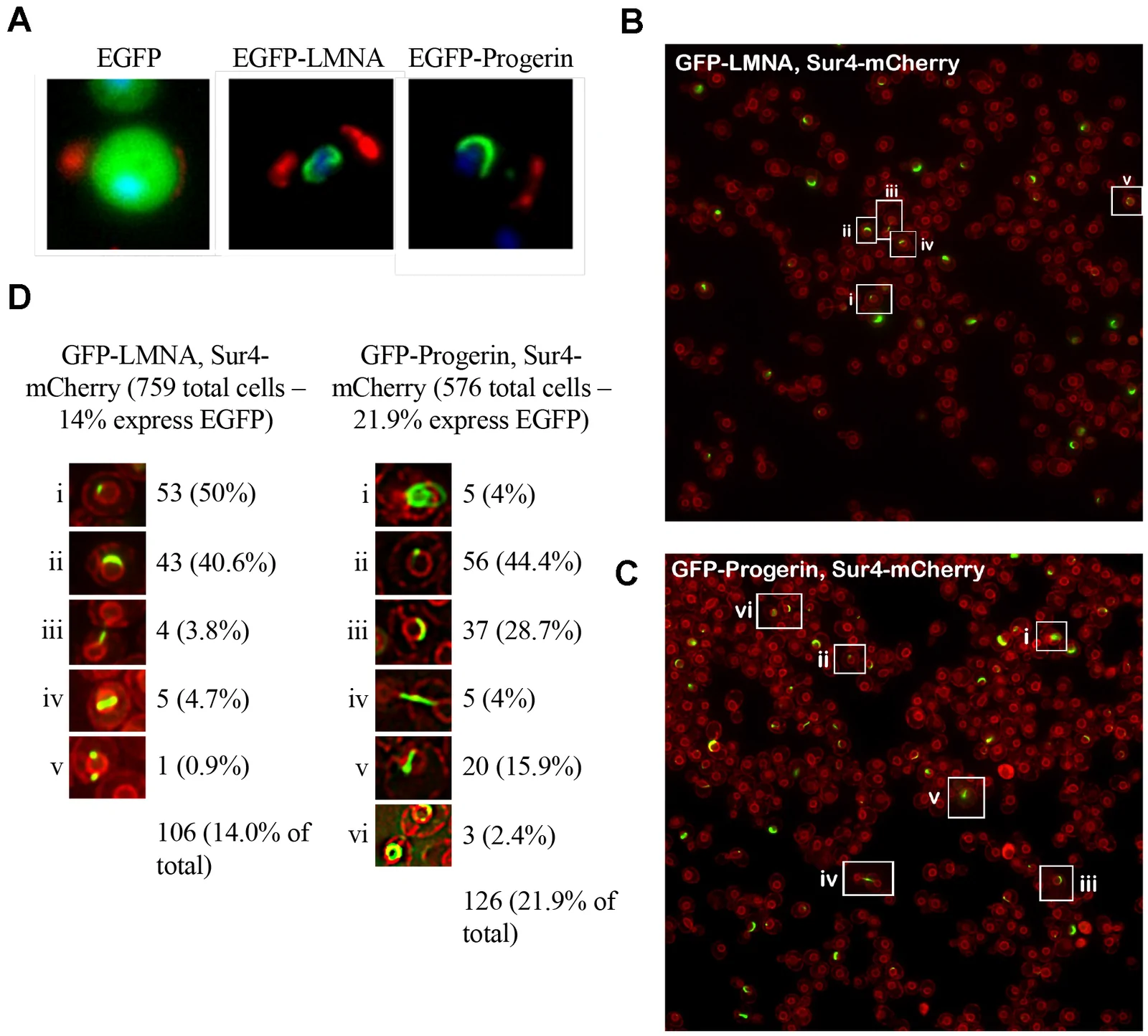
**EGFP-LMNA and EGFP-Progerin localize to the yeast nuclear membrane.** (**A**) Aliquots of cells from each EGFP (left panel), EGFP-LMNA (middle panel) and EGFP-Progerin (right panel) were fixed and prepared for fluorescence microscopy. Green signal indicates EGFP signal. Cells were labelled with wheat germ agglutinin (red) to mark bud scars on the cell periphery. Chromatin is counterstained with Hoechst 33342 dye to mark nuclei (blue). (**B**) and (**C**) Live cell imaging of fluorescence tags was performed in a yeast strain containing an endogenous *SUR4-mCherry* epitope to fluorescently mark the nuclear membrane (red). The *SUR4-mCherry* cells were transformed with EGFP-LMNA (**B**) or EGFP-Progerin (**C**), grown overnight in Ura- media supplemented with 2% Raf for 24h, diluted to an OD_600_ of 0.2 in fresh Ura- media supplemented with 2% galactose, then incubated at 30° C for 6 hours. (**D**) Examples of cells highlighted in (**B**) and (**C**) were shown in the inset regions, and counted. The percent of total cells expressing EGFP with different localization patterns is given.

To determine whether LMNA and Progerin were indeed associated with the nuclear membrane, we expressed EGFP-LMNA or EGFP-Progerin in Sur4-mCherry expressing cells to mark the nuclear periphery. We used live cell imaging and observed that EGFP-LMNA and EGFP-Progerin signals were associated with the Sur4-mCherry labelled nuclear membrane (Figure 2B, 2C). Cells with different EGFP nuclear membrane localization patterns were labelled in the wide field images shown in Figure 2B, 2C. The number of cells exhibiting these different patterns were counted and the percent of cells showing these morphologies was scored (Figure 2D). In EGFP-LMNA expressing cells (in 14% of cells), half of the EGFP positive cells expressed a single focus on the nuclear membrane (50% of the total cells with EGFP fluorescence). The next largest category was cells with a crescent shape along the nuclear membrane (43%). Smaller categories of other shapes were observed, but we did not observe LMNA completely encircling the nuclear membrane. Expression of EGFP-Progerin (in 21.9% of cells) caused similar features. Single foci (44% of cells) and crescent shapes (28.7% of cells) were observed the most often, similar to LMNA expressing cells. We observed EGFP-Progerin signal encircling the nucleus in 2.4% of cells. To determine if any localization patterns are associated with the kinetochore or nucleolus, we used Mtw1-mCherry and Nop56-mCherry cells, respectively, expressing EGFP-LMNA or EGFP-Progerin, but did not observe any colocalized signals (data not shown). Overall, we observed higher numbers of variants in EGFP-Progerin expressing cells that were associated with the nuclear membrane, compared to LMNA expressing cells, but the pattern differed from that observed with LMNA in human cells, which is typically localized to the nuclear periphery [14, 16].

### EGFP-Progerin is a stable protein that is not appreciably degraded in yeast cells

Our next objective was to determine the fate of these proteins in yeast cells. To study the catabolism of EGFP, EGFP-LMNA, and EGFP-Progerin, we induced protein expression by incubation for 4 h at 30° C in 2% RAF/0.2% GAL. Expression of recombinant proteins was terminated by resuspending cells in media supplemented with 2% glucose. Following exchange into media with glucose, culture aliquots were taken and subjected to Western blotting to monitor levels of EGFP, EGFP-LMNA, and EGFP-Progerin. We initially probed the breakdown of EGFP-LMNA and EGFP-Progerin by exchanging cells into glucose medium and treating them with cycloheximide to prevent new protein synthesis (Figure 3A). We observed no difference in levels of EGFP, EGFP-LMNA, or EGFP-Progerin after 60 minutes of treatment with cycloheximide. Longer treatments with cycloheximide did not result in observable decreases in protein levels of EGFP, EGFP-LMNA, or EGFP-Progerin, consistent with a previous report [37], and were associated with significant levels of cell death (data not shown). As such, we further probed the rate of protein degradation by Western analysis of protein extracts from cells after switching to glucose containing media over 24 hours (Figure 3B; quantified in Figure 3D). Upon the addition of glucose, the *GAL1* promoter is repressed, stopping the transcription of genes encoding EGFP-tagged proteins. Longer periods of protein stability can be viewed using this approach compared to CHX treatment. EGFP protein levels in control cells, following a switch to glucose, were lowered after 2 h (75 +/- 9%), decreased further after 4 h (45 +/- 4%), until reaching 0% after 24 h. Levels of EGFP-LMNA reached 63 +/- 4% of its starting quantity after 4 h and dropped to 30 +/- 5% after 24 h. Levels of EGFP-Progerin remained stable after 4 hours, with 46 +/- 6% of starting material left after 24 h. The data indicates that EGFP has a protein half-life of approximately 4 hours while EGFP-LMNA and EGFP-Progerin appear to have half-lives of greater than 4 hours when using this method.

**Figure 3:**
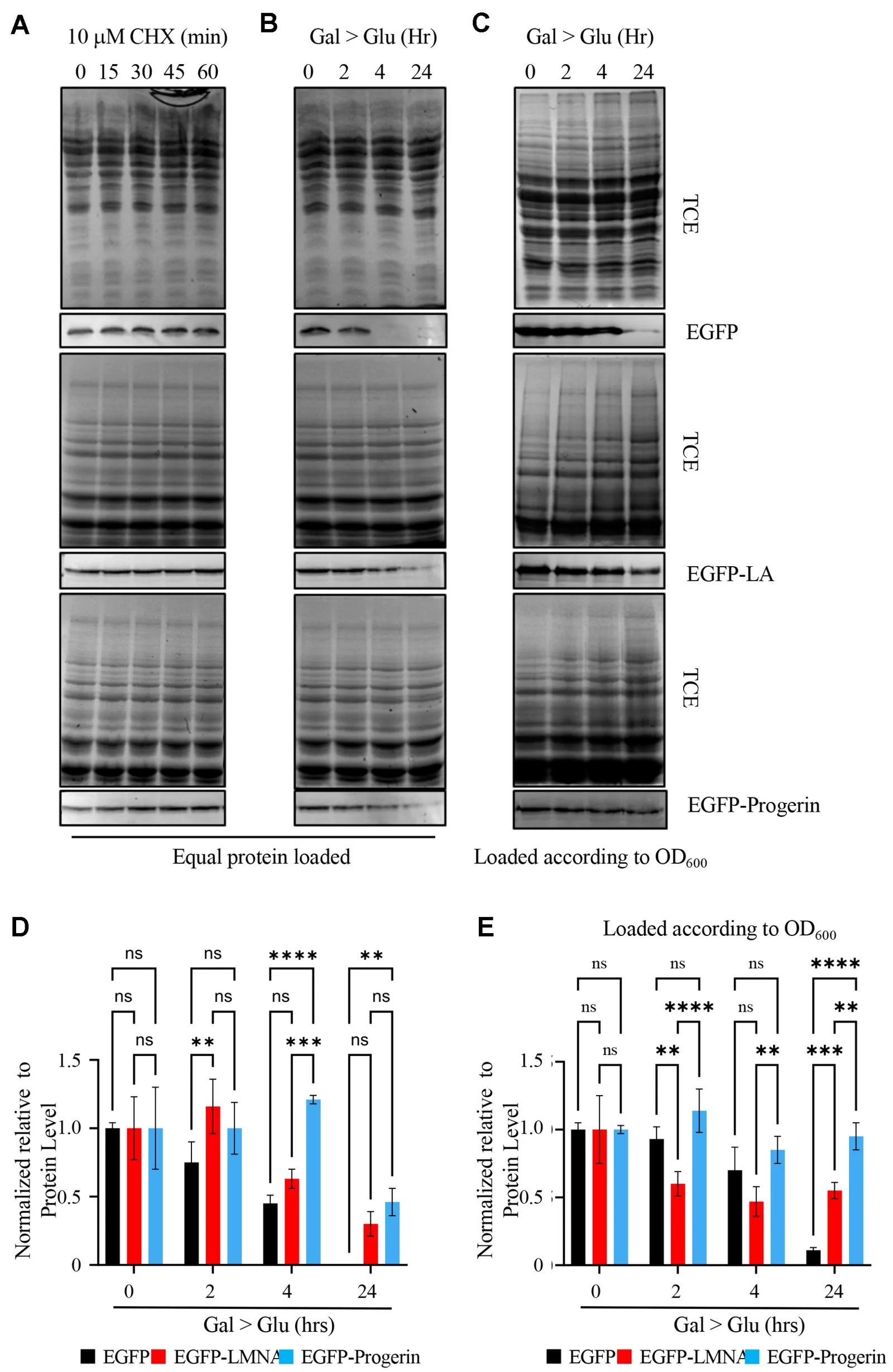
**EGFP-Progerin decreases in transformed cultures as a function of total protein but not as a function of cell numbers.** (**A**) To evaluate the fate of EGFP (upper row), EGFP-LMNA (middle row) and EGFP-Progerin (bottom row), we treated cultures expressing these proteins with cycloheximide for 0, 15, 30 and 60 min to determine if cells degraded these proteins. Over the time course, proteins were evaluated for total protein content by trichloroethanol (TCE) staining followed by Western blotting for the EGFP tag. (**B**) To further evaluate the levels of these proteins, we switched EGFP (upper row), EGFP-LMNA (middle row) and EGFP-Progerin (bottom row) cultures from induction media (GAL) to glucose (GLU) to repress the transgenes and followed the levels of EGFP tagged proteins over 0h, 2h, 4h and 24h. Total protein was isolated from each culture and equal protein amounts were loaded onto gels. Total protein was evaluated by TCE staining and Western blots performed to evaluate the level of EGFP tagged proteins. (**C**) Total protein was isolated from cell equivalents based on cell density with the culture and EGFP (upper row), EGFP-LMNA (middle row) and EGFP-Progerin (bottom row) evaluated by Western blot. (**D**) The experiment described in (**B**) was repeated 3 times, with bands scanned and quantified. SEM is shown. Two-way ANOVA with *post hoc* Tukey’s multiple comparisons test was used to determine p-values. (**E**) The experiment described in (**C**) was repeated 3 times, with bands scanned and quantified. SEM is shown. Two-way ANOVA with *post hoc* Tukey’s multiple comparisons test was used to determine p-values. p < 0.01**, p < 0.001***, and p < 0.0001****.

Considering that Lamin A and Progerin have poor solubility [37], assessing Lamin A and Progerin levels using protein purification methods is difficult. The 24-hour shut off experiment in glucose allows us to get a better view of protein stability over the long term. While we observed substantial decreases in levels of EGFP, EGFP-LMNA, and EGFP-Progerin after switching cells from raffinose/galactose to glucose containing media for 24 h in Figure 3B, 3D, we also noted that there was a 2-4-fold increase in cell density during the time cells were in glucose containing medium (data not shown). Given the increase in cell number after 24 h in glucose, we hypothesized that the reduced protein levels observed when loading equal quantities of total protein in the Western blots shown in Figure 3B (quantified in Figure 3D) might be the combination of decreased protein levels due to protein catabolism in addition to dilution of proteins due to cell division/increased cell numbers. For example, a cell doubling will dilute EGFP-tagged protein levels to 50%, which is independent of protein stability. We addressed this by loading Western blots with protein extracts from cells switched to glucose containing media in proportion to the culture density rather than based on equal total protein (Figure 3C; quantified in Figure 3E). This revealed that the total quantity of EGFP control protein decreased to 70 +/- 10% after 4 h and to 11 +/- 1% by 24 h. Total quantities of the EGFP-LMNA were lower than EGFP control levels after 2 (60 +/- 5%) and 4 h (47 +/- 6%), with the difference significant after 2 h, but did not reach a statistical value <0.05 at 4 h. However, the LMNA levels remained stable from 4 hours to 24 h (55 +/- 3%), opposed to control levels that dropped to 11%. Total quantities of the EGFP-Progerin, on the other hand, did not decrease observably over the 24 h release (114 +/- 9% (2 h), 85 +/- 6% (4h), 95 +/- 6% (24 h); Figure 3C; quantified in Figure 3E). This data shows that the quantity of the EGFP-Progerin fusion protein relative to total protein in Figure 3B decreased primarily due to dilution during cell growth and division. However, there is little, if any change in the total quantity of EGFP-Progerin when the dilution factor is eliminated, indicating it is not appreciably degraded in yeast cells under these conditions.

To further examine the fate of the EGFP, EGFP-LMNA, and EGFP-Progerin fusion proteins during cell growth and division, as well as to confirm our previous observation from Western blotting (Figure 3), we used fluorescence microscopy to analyze EGFP signals once cells were shifted into glucose-containing media. Cells were also labelled with wheat germ agglutinin (WGA; red) to identify bud scars and counterstained with H33342 (blue) to identify nuclei. Our initial analysis of images (Figure 4A) shows yeast cells expressing EGFP, EGFP-LMNA, or EGFP-Progerin after 4 h in 2% RAF/0.2% GAL and after a 4 and 24 h shift into media containing 2% glucose to shutoff the GAL inducible promoter. After 4 h in medium containing GAL, most control cells showed bright and diffuse EGFP signal distributed evenly throughout the cytoplasm, while after 24 h in glucose-supplemented medium, only faint EGFP signal could be detected. Cells expressing EGFP-LMNA or EGFP-Progerin showed many cells with bright EGFP signal after a 4 h incubation in GAL media, as expected. The images (Figure 4A) revealed that the EGFP-LMNA and EFGP-Progerin fluorescence is smaller and more defined than that observed with EFGP, and localized to the nucleus. The WGA staining indicates that young (one or few bud scars) and old (many bud scars) cells appear to equally express the fusion proteins after a 4 h induction. After 4 and 24 h in media containing glucose, we observed that there were considerably fewer cells with EGFP signal (Figure 4A). The percentage of cells expressing the constructs after induction and promoter shutoff is shown in Figure 4B. At time 0 of promoter shutoff (immediately following 4 hours of induction), 85% of EGFP, 45% of EGFP-LMNA, and 50% of the EGFP-Progerin transformed cells demonstrated fluorescence. After 4 h of promoter shutoff, the percentages dropped to 55%, 35%, and 25% and after 24 h the percentages dropped to 9%, 12% and 18% for the EGFP, EGFP-LMNA and EGFP-Progerin, respectively. However, when we compared the intensity of the signal inside single cells positive for GFP signal (normalizing intensity to 1 at 0 h in all 3 cultures), cells expressing EGFP alone exhibited low values after 24 h of promoter shutoff. The intensity of EGFP-LMNA and EGFP-Progerin were higher in single cells with green fluorescence, with the intensity of the EGFP signal in individual EGFP-LMNA and EGFP-Progerin positive cells following 24 h of promoter shutoff comparable to that of cells following 4 h induction (Figure 4C). The data demonstrates that EGFP-LMNA and EGFP-Progerin fusion proteins exhibit increased stability compared to EGFP alone, with mean EGFP signal intensity in EGFP cells decreasing to 13 ± 9% of its original value after 24 h while EGFP-LMNA and EGFP-Progerin signal intensity decreased to 76 ± 13% and 81 ± 16% of their initial values, respectively. Overall, our initial analyses indicated that shifting cells to glucose to shutoff the galactose inducible promoter resulted in fewer cells with detectable EGFP signal, but in cultures expressing the EGFP-LMNA and EGFP-Progerin fusion proteins, these cells showed similar EGFP signal intensity as cells grown in RAF/GAL containing media. Overall, the data in Figure 4A–4C show that yeast cells target the EGFP protein for degradation, whereas EGFP-LMNA and EGFP-Progerin fusion proteins remain relatively stable after 24 hours, similar to that observed in Figure 3.

**Figure 4:**
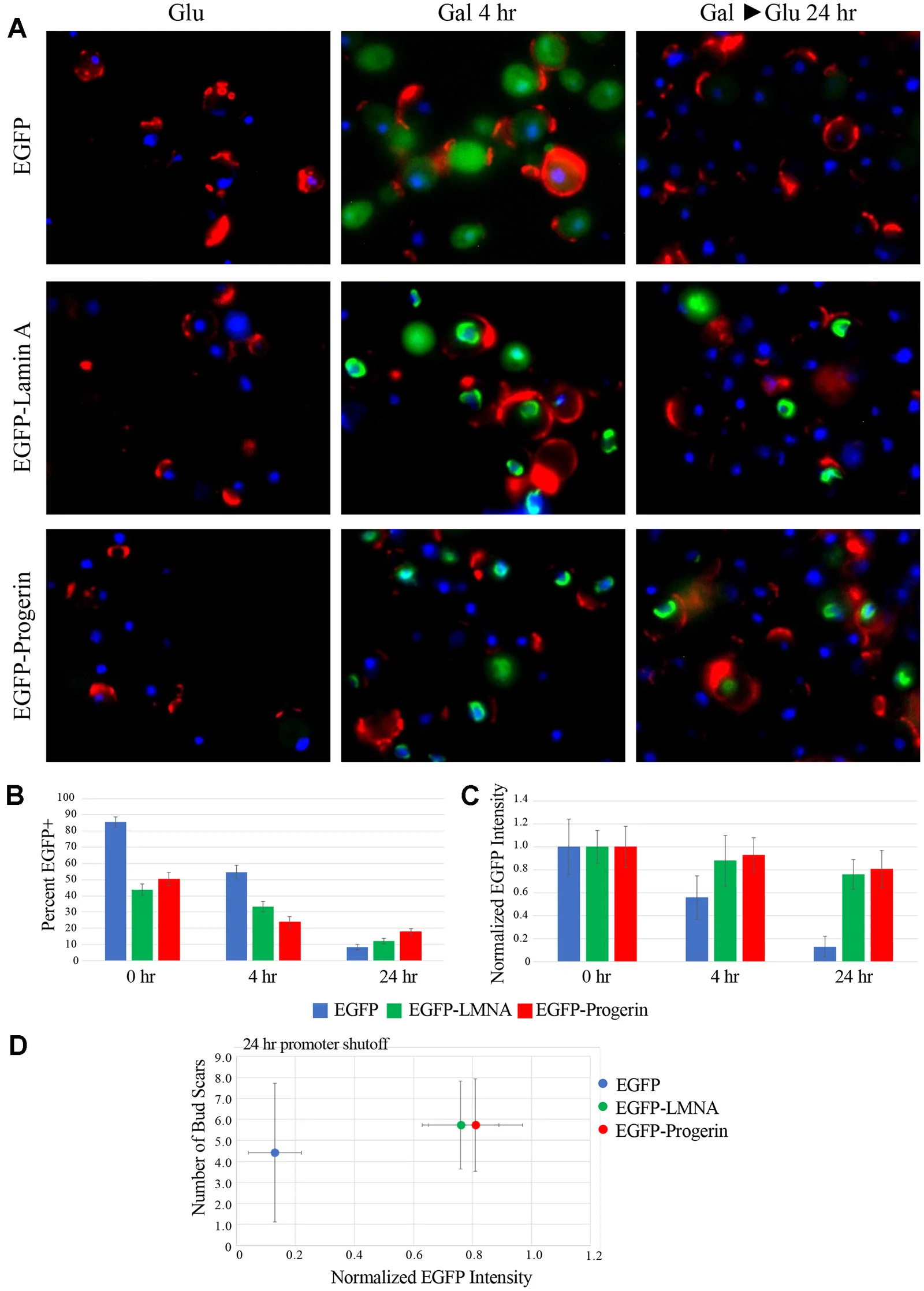
**EGFP-LMNA and EGFP-Progerin are maintained at high levels in a subpopulation of cells following removal of galactose induction.** (**A**) Aliquots of cells were taken from EGFP (upper row), EGFP-LMNA (middle row) and EGFP-Progerin (bottom row) at under either pre-induction (GLU) conditions, following 4h of induction (GAL 4H) or 24 of removal from induction (GAL>GLU 24H). Cells were fixed and labelled with WGA (red), DNA counterstained with H33342 (blue) and EGFP signal is shown in green. (**B**) Micrographs of fixed and labelled cells from EGFP (blue bars), EGFP-LMNA (green bars) and EGFP-Progerin (red bars) were quantified. The percentage of positive cells from each culture after induction and moving the cells back into glucose containing media following 0h, 4h and 24h were scored as a percentage (y-axis). (**C**) From the positive cells from each of the EGFP (blue bars), EGFP-LMNA (green bars) and EGFP-Progerin (red bars), the intensity levels of signal were measured at 0h, 4h and 24h. Data is normalized to the 0h time point. Images were collected from n=3 cultures of each EGFP, EGFP-LMNA and EGFP-Progerin. n=200 cells for each. Error bars represent SEM. (**D**) EGFP-expressing (blue), EGFP-LMNA expressing (Green), or EGFP-Progerin expressing (red) cells analyzed in Figure 3A were further analyzed by measuring the total intensity of green fluorescence in each cell. The average number of bud scars in each population of cells is plotted on the Y-axis while the average normalized EGFP fluorescence is plotted on the X-axis. Error bars represent the SEM.

### EGFP-Progerin is preferentially retained in older yeast cells

To quantify the amount of protein in transformed cells as a function of cell age, we analyzed the images shown in Figure 4A to determine if there was a relationship between EGFP-LMNA and EGFP-Progerin expression with age. These cells were labelled with fluorescently tagged WGA to measure the number of bud scars present on each cell. Bud scars are areas of exposed chitin left when daughter cells bud from their progenitor mother cells, with the exposed chitin recognized by WGA [38]. Counting bud scars is a qualitative measure to indicate differences microscopically between young and old cells. Over the course of a 24 h period, we interpret cells that have higher numbers of bud scars as replicative older than those with fewer. We acknowledge that a cell with 5 bud scars is still relatively young; however, we previously demonstrated that yeast aging begins as early as 2 divisions [39]. As such, we consider cells with at least 5 bud scars to be aging. 200 cells at each time point were randomly selected and scored manually as to whether EGFP signal was present and, for those cells with detectable EGFP signal, the sum of the pixel intensity in the EGFP channel was measured (Figure 4D). WGA signals were also imaged to track the relative age of each cell. The average normalized fluorescence intensity of cells with detectable EGFP alone was sharply reduced by 24 h, providing quantitative evidence for our visual observations. In contrast, while the number of EGFP-LMNA or EGFP-Progerin expressing cells decreased over time, the per-cell intensity remained high after 24 hours (Figure 4D). Taken together, our data demonstrates that while the numbers of cells expressing the fusion proteins decreased following promoter shutoff, the quantity per cell remained relatively stable.

To further assess the relationship between EGFP-Progerin and older cells, we developed a methodology that would enable rapid analysis of larger numbers of cells for fusion protein content in aging cells using flow cytometry of formalin-fixed yeast cells stained with fluorescently labelled WGA. The use of flow cytometry to detect WGA fluorescent protein content in yeast has been described previously [40, 41], but to our knowledge, this is the first instance of using bud scars labelled with fluorophore-conjugated WGA in flow cytometry to measure the age of the population. We, therefore, first set out to demonstrate the effectiveness of this technique by comparing flow cytometry data of untransformed wild-type cells after 2 h and after 24 h of culture (Figure 5A). The intensity of WGA staining of birth scars on virgin daughters is much less than the intensity of bud scars staining on mothers [42], therefore, we expect that WGA staining should increase in the culture as the cells replicative age. Indeed, the data show that the average AlexaFluor®-555 conjugated WGA (WGA-AF555) fluorescence of the density of the cell population increased over the time course, as expected. We next tested the ability of flow cytometry to measure differences in EGFP signal content concomitantly with cell age with AF555-WGA. Cells expressing the control EGFP tagged proteins were fixed and stained with AF555-WGA, either before, or after 4 hours in inducing media with data plotted as AF555-WGA-fluorescence versus EGFP fluorescence (Figure 5B). The data shows a pronounced increase in the number of cells with increased EGFP fluorescence during incubation. Taken together, these data support the use of flow cytometry to monitor the interactions between cell age and EGFP fusion proteins metabolism in yeast.

**Figure 5:**
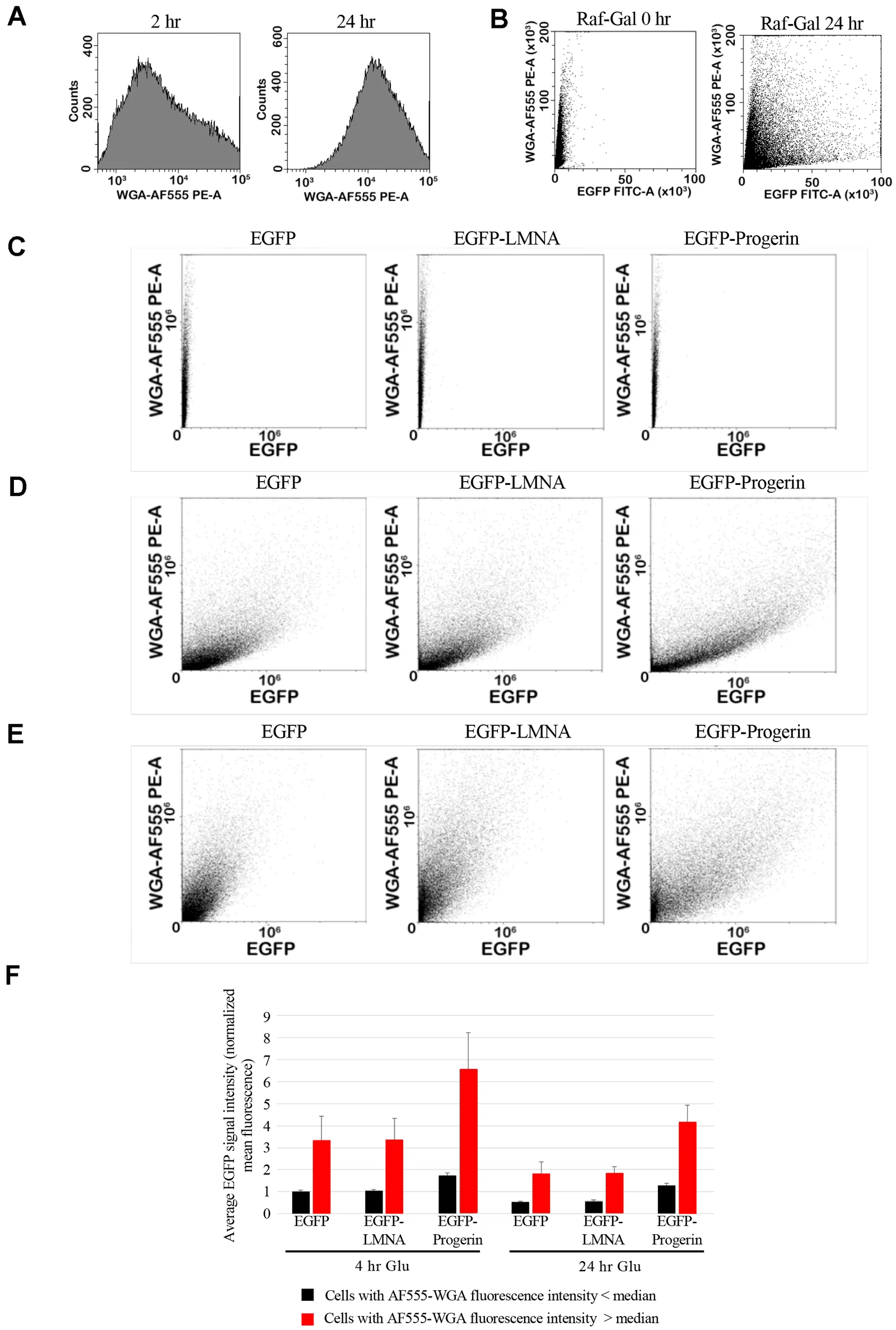
**EGFP and bud scar signal can be measured by flow cytometry.** (**A**) To quantify bud scar signal by flow cytometry, cells were fixed and labelled with WGA conjugated to Alexa-555 dye (WGA-AF555). Representative histogram of the cell counts (y-axis) from untransformed cultures against intensity of label at 2h and 24 hours. The shift in the histogram represents increased average signal as the cultures age, correlated with higher average numbers of bud scars per cell, and therefore higher WGA-AF555 labelling. (**B**) Cells from cultures either immediately prior to induction (RAF GAL-0H) or after 4h incubation in RAF/GAL induction medium (RAF GAL-4H) were fixed and labelled with WGA-AF555. Flow cytometry was used to quantify EGFP signal (EGFP-FITC-A) and the WGA-AF555 signal (WGA-AF555-PE-A), demonstrating the detection of cells with higher levels of EGFP expression at the right of the panel and cells with higher intensity of bud scar labelling at the top of the panel. (**C**–**E**) EGFP (left column), EGFP-LMNA (middle column), and EGFP-Progerin (right column) expressing yeast were fixed for flow cytometry analyses; (**C**) prior to induction of fusion protein expression, (**D**) after 4 h of induction, and (**E**) and after 24 h incubation in media containing glucose following induction. Y-axis represents the intensity of WGA-AF555 signal, and the x-axis represents the intensity of EGFP signal. (**F**) Statistical data from the flow cytometry shown in panel A was used to analyze the average EGFP intensity of cells either immediately following 4 h induction in RAF/GAL medium (EGFP-0H-GLU, EGFP-LA-0H-GLU, and EGFP-D50-0H-GLU) or after 24 h in glucose containing medium (EGFP-25H-GLU, EGFP-LA-24H-GLU, and EGFP-D50-24H-GLU). Average EGFP signal intensity (normalized mean fluorescence) of younger cells showing less than the median WGA-AF555 intensity (black bars) or in older cells exhibiting greater than the median WGA-AF555 signal (red bars) was plotted. Error bars represent the standard error of the mean.

To examine in detail the relationship between cell age and fusion protein content, we used flow cytometry to analyze cultures of EGFP, EGFP-LMNA, and EGFP-Progerin expressing yeast prior to induction of protein expression, after 4 h of expression, and after 24 h incubation in media containing glucose to shutoff the inducible promoter (Figure 5C–5E). Prior to induction of EGFP and the EGFP-fusion proteins, cultures exhibited a range of WGA fluorescence intensities corresponding to a range of cellular ages (Figure 5C, Y-axes), while cells had uniformly low EGFP fluorescence (Figure 5C, X-axes). After 4 h post induction the distribution of AF555-WGA fluorescence was similar as prior to induction (Figure 5D, Y-axes), whereas all three cultures showed large numbers of cells with increased EGFP fluorescence (Figure 5D, Y-axes). It appears from the upward sweep of the plots that cells with higher levels of bud scars accumulate EGFP signals in all three transformed sets of cells, but overall, there does not appear to be a strong relationship between cell age and the expression of EGFP or the EGFP-LMNA and EGFP-Progerin fusions after 4 h of induction. However, following 24 hours of promoter shutoff, all three cultures exhibited a shift in the cell population to the left indicating overall reductions in the number of cells expressing EGFP and the EGFP-fusions (Figure 5E). None of the three cultures had the distributions of cells expressing EGFP or EGFP-fusions return to the pattern observed prior to induction, consistent with Western and fluorescence microscopy observations in which cells did retain EGFP or the EGFP-fusions following promoter shutoff. All three cultures demonstrated an apparent relationship between cell age and the residual EGFP signal, with younger cell populations near the bottom of the graph exhibiting lower EGFP signal than older cells located higher on the Y-axis (Figure 5E). In EGFP and EGFP-LMNA expressing cultures, there was a substantial reduction in cells retaining high levels of EGFP signal, while in EGFP-Progerin expressing cultures the population of cells which retained high levels of the fusion protein was increased, and many of these cells were also older since they exhibited higher AF555-WGA fluorescence (Figure 5E). These detailed observations employing flow cytometry clearly illustrate the relationship between cell replicative age and retention of the EGFP fusion proteins and show important differences in this effect between EGFP-LMNA and EGFP-Progerin expressing cells. Although EGFP-LMNA is retained by older cells, compared to EGFP alone, there was a marked increase in the number, age, and extent to which cells retain the EGFP-Progerin fusion protein.

Finally, we quantified these effects using the large datasets acquired through use of flow cytometry. Triplicate cultures were incubated in inducing media for 4 h and aliquots removed for fixation and analysis, and the remaining (unfixed) culture then exchanged into glucose containing media for a further 24 hours and fixed. These were subjected to flow cytometry and per-cell fluorescence intensity in both the AF555-WGA and EGFP channels were collected. Cells were divided into two groups, those below the median AF555-WGA fluorescence intensity (“young” cells) and those above the median AF555-WGA fluorescence intensity (“old” cells). The mean EGFP fluorescence for each group was then calculated and values derived from triplicate samples were combined. Data was normalized to the mean fluorescence intensity of EGFP-expressing control cells prior to exchange into glucose containing media (Figure 5F). After 4 h of induction, the mean fluorescence intensity of both young and old cells was similar in both EGFP and EGFP-LMNA expressing cells, with the older cells showing ~3 times higher average EGFP fluorescence than younger cells. In contrast, both young and old EGFP-Progerin expressing cells exhibited higher average EGFP fluorescence than the corresponding EGFP and EGFP-LMNA expressing cells (Figure 5F). After 24 h in glucose-containing media, all cell populations exhibited lower average EGFP fluorescence than at the end of induction. The average fluorescence intensity of both young and old cells expressing EGFP and EGFP-LMNA was similar after 24 h in culture, in contrast to the measurements obtained using densitometry of fluorescence micrographs and illustrating the advantage in sensitivity gained through use of flow cytometry. Fluorescence intensity decreased by 45% in both young and old cells expressing EGFP and by 43% in young and 45% in old cells expressing EGFP-LMNA, showing that both young and old cells can catabolize and eliminate these proteins as assessed by flow cytometry. For EGFP-Progerin expressing cells, young cells exhibited a 26% reduction and old cells showed a 36% reduction in average EGFP fluorescence, indicating that clearance of EGFP-Progerin is impaired in both old and young cells relative to EGFP-LMNA or EGFP alone. While flow cytometry data shows that both young and old cells are capable of clearing the EGFP-LMNA and EGFP-Progerin proteins, the data also shows that older cells contain greater quantities of these proteins, and moreover, that levels of EGFP-Progerin are much higher than levels of EGFP-LMNA in older cells. Currently the significance of higher levels of EGFP-Progerin in older cells is unclear and may be a product of the previously established phenomenon that older cells are less capable of protein turnover than younger cells [19, 39, 43, 44]. Furthermore, yeast mother cells have been shown to retain toxic molecular aggregates from their daughter cells [45]. This provides further evidence that Progerin, but not necessarily LMNA expression in yeast, is considered toxic and must be asymmetrically retained from the daughter cells to ensure the daughters are born young. Nevertheless, the data show that, in contrast to observations made with fluorescence microscopy alone, yeast cells can effectively catabolize and eliminate EGFP and EGFP-LMNA with similar efficiency in both old and young cells, while EGFP-Progerin is more effectively retained in older cells. In addition, the data do show that some catabolism of EGFP-Progerin is occurring, even in old cells, indicating that yeast is potentially a useful model system for studying the pathways by which EGFP-Progerin is broken down and for potential therapeutic interventions aimed at accelerating this process.

## DISCUSSION

A major driver of cellular aging is the accumulation of stable proteins that significantly disrupt cellular function. In mammalian cells, the most extreme example of this is seen in patients suffering from the premature aging disease, Hutchinson Gilford Progeria Syndrome (HGPS). Progerin, the molecular determinant of HGPS, is a cytotoxic protein that results from a point mutation within the *Lamin A/C* gene and accumulates in the nuclear lamina, disrupting genome function and organization. For research purposes, this protein makes an excellent case study of how protein accumulation can have deleterious impacts on cellular health and lifespan. As such, our overarching goal was to develop a novel model that incorporates Progerin in yeast in order to rapidly test and screen compounds or strategies that promote clearance of these cytotoxic protein accumulations as well as to study the fate of these proteins.

Although not identified as members of the lamin family, early yeast research identified proteins that reacted with antibodies raised against avian and mammalian lamin A and B, but not C, proteins in yeast [46]. However, these are not intermediate filament proteins and as such are not true lamin proteins. These lamin-like proteins were part of the nuclear fractions, indicating that there is a similar structure or at least series of proteins that make up the yeast nuclear periphery; however, further information on the identify of these proteins was not available. Although yeast have a closed mitosis, these observations do indicate that there is some parallel or distant relationship between the yeast nuclear periphery and that found in higher eukaryotes. In 1995, it was demonstrated that mammalian lamin proteins, including prelamin A, LMNA, lamin B1 and lamin C could be expressed in ‘humanized’ yeast [47]. Yeast models such as this have been used to study the processing of prelamin A to lamin A and have become a model to evaluate drugs [48]. Additionally, yeast have been used as a model to study the processing of prelamin when co-expressed with the metalloprotease ZMPSTE24 [49], which cleaves prelamin A at the C-terminal end of the protein, within the region that is deleted in Progerin. Mutations in the ZMPSTE24 sequence phenocopy that of Progerin expression in HGPS [48].

By expressing either EGFP, EGFP-tagged human lamin A (EGFP-LMNA) or EGFP-Progerin under the control of a galactose inducible promoter, we identified that yeast cells direct both of these fusion proteins to the nuclear membrane (Figure 2). Not only did the proteins localize to the correct location, EGFP-Progerin significantly decreased cell growth and chronological lifespan, and increased genomic instability (Figure 1C–1E), parallel to what is observed in mammalian cells. We used a yeast colony sectoring assay to identify a defect in genome stability, where cells expressing EGFP-LMNA, and EGFP-Progerin to a higher degree, exhibited a red to white colony reversion resulting when the red color produced as a result of the point mutation in the *ADE2* gene is reverted to white following any mutation that restores or bypasses the *ade2-101_ochre* allele, as we have previously shown [35, 36]. We observed that both EGFP-LMNA and EGFP-Progerin caused increased rates of colony reversion, a direct readout of genome instability. Progerin and LMNA both induced significant increases in genomic instability at all time points compared to the EGFP control (Figure 1E). However, EGFP-Progerin generated a higher rate of genomic instability than EGFP-LMNA. Progerin in mammalian cells is well known to cause DNA damage, epigenetic change, changes in transcriptional readouts, all contributing to genomic instability. In mammalian cells, defective Lamin A accumulates and sequesters replication and repair components, such as PCNA, causing replication forks to stall and collapse into double strand breaks [34, 50]. We do not yet know if this occurs in yeast, but it is clear that Progerin, and LMNA to a lesser degree, increase genomic stability. This assay demonstrates that yeast expressing Progerin is an excellent model for testing compounds that mediate protein accumulation or measure the impact of ectopic expression of proteins on genome stability. Given the phenotypes of decreased cell growth, reduced chronological lifespan, and genome instability, we conclude that our yeast model will make an excellent model to study the impact of cytotoxic protein accumulations.

The increase in genomic instability caused by EGFP-LMNA may be explained by the accumulation of this protein in the nuclear periphery. It is well documented that the accumulation of excess LMNA protein phenocopies Progerin. Deletion of the gene that encodes the ZMPSTE24 metalloprotease that cleaves the C-terminal end of LMNA results in pre-lamin A accumulation in the nuclear lamina in mammalian cells [51]. Given that previous research into the expression of LMNA along with the expression of mutant ZMPSTE24 demonstrate progeroid phenotypes in yeast, it is logical that we observed parallel results and that expression of EGFP-LMNA has impacts on yeast genome stability. However, yeast appear to be better equipped to deal with LMNA compared to Progerin, as seen through the greater impact of EGFP-Progerin on genome instability. This is also supported by our other results indicating that GFP-LMNA does not have significant impacts on other hallmarks of aging compared to GFP-Progerin, as seen in chronological lifespan assays.

We observed that EGFP-LMNA and EGFP-Progerin decrease in the population after the removal of galactose to induce ectopic gene expression as predicted. To shut off the expression of the EGFP fusion proteins, we grew the cells in glucose supplemented media for up to 24 hours following a 4-hour induction of the proteins in 0.2% galactose. According to our western blot analysis (Figure 3B, quantified in Figure 3D), we observed that the fusion proteins decreased in the population over time. However, the proteins were being diluted out of the population due to cell doubling, with no new production of the fusion proteins. To determine if the protein reductions are simply due to dilution, we loaded cell equivalents according to OD_600_ readings over the 24-time course, onto gels for Western analyses and identified a much slower decrease in EGFP-LMNA and no significant decrease in EGFP-Progerin (Figure 3C, quantified in Figure 3E). We further used fluorescence microscopy to examine the population of cells that expressed EGFP-LMNA and EGFP-Progerin. Following the removal of galactose, we observed that EGFP-LMNA and EGFP-Progerin were not being degraded but were retained in a sub-population of cells (Figure 4A, quantified in Figure 4B, 4C). This sub-population had intensity levels of fluorescence comparable to that of cells that were induced with galactose (Figure 4C), indicating that the yeast were not turning these proteins over, but retaining them. To further identify what population cells maintaining high levels of EGFP-Progerin were derived from, we labelled cells with fluorescently tagged wheat-germ agglutinin (WGA), which binds to the bud scars on the surface of yeast mother cells, and determined that the population that retained EGFP-Progerin had high numbers/intensity of WGA (Figure 4D). We interpreted this as the cells retaining high levels of EGFP-LMNA and EGFP-Progerin were those that had an ‘older’ replicative lifespan.

To further characterize this observation, we developed a novel high-throughput assay using flow cytometry to measure the amount of EGFP-signal from fusion proteins as well as signal from bud scars via WGA labelling (Figure 5). We found, using flow cytometry to measure WGA fluorescence, that WGA levels rise after 24 hours of growth (Figure 5A). It is possible to measure WGA fluorescence in aging cells since WGA stains the birth scars of virgin daughters less intensely than bud scars on mother cells [42]. This is the first time, to our knowledge, that flow cytometry of WGA has been used to measure cell aging, although flow cytometry has been used in the past to measure WGA staining [41]. Following induction, EGFP signals increased quickly within the population (Figure 5B). Following removal of galactose, with the addition of glucose, a subpopulation of cells that retained high EGFP signal was also observed to have higher levels of signal for the WGA label (Figure 5D, 5E). The quantitation performed identified that EGFP-Progerin, specifically, was retained in older yeast cells, compared to EGFP and EGFP-LMNA (Figure 5F). The conclusions from these observations reflected those from microscopic analyses and corroborated those from using cell equivalents. In addition, this method provides a new avenue to examine yeast replicative lifespan in relation to tagged protein expression; a tool with broad applications in the field of yeast and aging research.

Our interpretation of the above observations is that the mother cells are retaining these cytotoxic proteins, in particular EGFP-Progerin, to keep daughter cells in a pristine state. Examination of previously published literature indicates that EGFP-Progerin and, to a limited extent, EGFP-LMNA fall into a category of molecules called long-lived asymmetrically retained proteins (LARPs) [52]. The hypothesis indicates that mother cells will retain LARPs to prevent the inheritance of these proteins in daughters. Although it is stated that asymmetrical localization and cell polarity play a role in the retention of LARPs the mechanisms surrounding this are not well defined. Regardless, our observations support this hypothesis, with EGFP-Progerin and EGFP-LMNA being retained in cells that have higher numbers of bud scars, thus protecting future generations of yeast cells from the cytotoxic effects of Progerin.

## CONCLUSION

Our main conclusion is that yeast expressing EGFP-Progerin is an excellent translational model for studying aging, and for the potential of testing compounds or strategies aimed at stimulating cells to degrade cytotoxic protein accumulations to enhance healthspan, in particular those in the nuclear periphery. We conclude that EGFP-Progerin acts as a long-lived asymmetrically retained protein that promotes genome instability and negatively impacts cellular lifespan. Finally, we conclude that our newly developed flow cytometry methodology for measuring EGFP tagged proteins in combination with fluorescently labelled WGA is excellent for the evaluation of protein retention and replicative lifespan.

## MATERIALS AND METHODS

### Vector construction, strains, media, and culture conditions

The human Lamin A and Progerin coding sequences were subcloned from pcDNA3.1 using the BglII and EcoRI restriction sites into pEGFP-C1 (Invitrogen) to generate pEGFP-LMNA and pEGFP-Progerin. The EGFP, EGFP-LMNA, and EGFP-Progerin coding sequences were amplified by high fidelity PCR using primers incorporating *attB* sites and the resulting products were cloned first into pDONR221 and then pYES-DEST52 using the Gateway® cloning system (Invitrogen). These procedures provided the pYES-EGFP, pYES-EGFP-LMNA, and pYES-EGFP-Progerin vectors used here for expression of the encoded proteins in *Saccharomyces cerevisiae* under control of the *GAL1* promoter. Full vector sequences and maps are available upon request.

Strains used in this study were BY4741 (S288C MAT*a his3Δ1 leu2Δ0 met15Δ0 ura3Δ0*) and YPH500 (S288C MAT*α ura3-52 lys2-801_amber ade2-101_ochre trp1Δ63 his1Δ200 leu2Δ1*). The Sur4:mCherry strain was constructed in the BY4741 background. The *mCherry-NAT* cassette was integrated just before the *SUR4* stop codon as previously described [53]. Unless otherwise stated, cultures were maintained in 1% yeast extract, 2% peptone, and 2% dextrose supplemented media (YPD) at 30° C. Rich Selective Medium (RSM) and Minimal Selective Medium (MSM), both lacking uracil, were prepared according to Malo et al. [36]. Transformations used the Lithium Acetate-PEG method [54].

The following standardized protocol that was used in this work utilized single colonies from agar plates that were inoculated into 3 mL of RSM containing 2% w/v glucose (RSM 2% GLU) and incubated for 18 h at 30° C and then poured into 50 mL RSM 2% GLU and incubated an additional 24 hours at 30° C. Cultures were centrifuged (5000 X G, 5 min) and resuspended in 50 mL pre-warmed (30° C) RSM 2% GLU and incubated 2 h at 30° C. For expression, cultures were then centrifuged again (5000 X G, 5 min), resuspended in 50 mL prewarmed RSM containing 2% w/v raffinose and 0.2% w/v galactose (RSM 2% RAF 0.2% GAL), and incubated 4 h at 30° C. We determined that the addition of 0.2% galactose to 2% raffinose induced EGFP-LMNA and EGFP-Progerin in 4-6 hours that still allowed reasonable growth that was not toxic. Half-lives of expressed proteins were analyzed by centrifugation of expression cultures and resuspending cells in pre-warmed RSM 2% GLU.

### Growth curves, chronological lifespan assays, and genomic instability assays

Growth curves and chronological lifespan assays were performed in strain BY4741. Time point 0 was set to 1 (100%) and then all subsequent timepoints were = t(x)/t(0). For plotting the logs from growth analyses, the value is the log of the normalized value, making the data directly comparable on the graph. Chronological lifespan (CLS) curves were constructed from triplicate cultures by removing small aliquots at the indicated time points and combining with the methylene blue yeast vitality reagent [55], then counting the number of viable (white) cells with a hemocytometer. CLS assays were conducted by growing cells for 18 h in RSM 2% GLU, centrifuging, and resuspending cells in 10 mL MSM containing 2% w/v raffinose and 0.2% galactose (MSM 2% RAF 0.2% GAL), incubating at 30° C until less than 10% was viable, while removing daily aliquots for determination of viable white cells. Performing the assay in MSM resulted in a shorter, more manageable CLS [34]. CLS was also performed by plating aliquots of cultures on YPD and counting the resulting colonies (Supplementary Figure 2), as previously described [36], which gave essentially identical results to those obtained using methylene blue vital staining and cell counting. Statistical analysis used a two-tailed t-test with p-values less than 0.05 being considered significant.

Genomic instability assays relying on the previously described red-and-white colony assay [36] were performed in strain YPH500, as this strain harbored the *ade2-101_ochre* allele, allowing for red/white colony sectoring. Cells were grown in RSM 2% GLU 18 h at 30° C and then collected by centrifugation and resuspended in 10 mL RSM 2% RAF/0.2% GAL. Cultures were incubated at 30° C with aliquots removed daily. The cells were diluted to ~100 colonies/20 mL and plated on YPD media, with the number of red and white colonies counted manually. Statistical analysis used a two-tailed t-test with p-values less than 0.05 being considered significant.

### Protein extraction and western blotting

Protein extraction was performed using a method based on previously described sodium hydroxide-based protocols with significant modifications [36]. For protein extraction 5 – 10 mL of culture was centrifuged (5000 X G, 5 min) and the resulting cell pellet resuspended in 500 μL of ice-cold 0.5 M sodium hydroxide and incubated on ice for 10 minutes. Samples were centrifuged (13,000 X G, 5 minutes, 4° C) and supernatants removed by aspiration. Pellets were resuspended in 1 X SDS-PAGE loading buffer containing 50 mM DTT and frozen at -20° C. Prior to loading, samples were heated at 80° C for 10 minutes, chilled on ice, and centrifuged (13,000 X G, 5 min).

Equal quantities of extracted proteins were resolved on polyacrylamide SDS-PAGE gels containing trichloroethanol (TCE) and transferred to PVDF for Western blotting according to standard protocols. Samples expressing EGFP-LMNA and EGFP-Progerin were resolved on 8% SDS-PAGE gels while samples expressing EGFP were resolved on 12% gels. Prior to blotting gels were imaged using UV light for detection of total protein via TCE-induced fluorescence. Antibodies used were mouse anti-GFP monoclonal (Protein Tech) at 1:10,000 and goat anti-mouse HRP conjugate (Protein Tech) at 1:10,000. Blots were imaged using enhanced chemiluminescent reagent.

For loading of cell equivalents instead of protein equivalents and to compensate for cell dilution, we performed the following loading: for example, if 20 uL of extract from a culture at t=0, OD_600_ = 1.0, was loaded, then at t = 1h, if the OD of that same culture was 1.5, then 1.5 X 20 = 30 uL of extract was loaded. The central theme here is that the protein present in that first 20 uL is diluted by cell division, so increasing the loading proportionally to the increase in OD ensures we compensate for dilution and are only observing changes due to degradation.

### Fluorescence microscopy, image analysis, and flow cytometry

For fluorescence microscopy and flow cytometry, samples were removed from yeast cultures as indicated and combined with 1/10 volume of formalin (37% w/v formaldehyde, 10% v/v methanol) and incubated for 10 minutes at RT on a tube rotator. Fixed cells were collected by centrifugation (5,000 X G, 5 min, RT) and supernatants removed by aspiration. Pellets were resuspended in 1 mL of distilled water containing 0.1% w/v sodium azide and stored in the dark at 4° C prior to further processing. Aliquots of fixed cells were collected by centrifugation (5,000 X G, 5 min, RT) and resuspended in tris-buffered saline (TBS, 50 mM Tris-HCl pH 7.2, 150 mM NaCl). Alexafluor-555 conjugated wheat germ agglutinin (WGA-AF555, ThermoFisher, Cat # 32464) and Hoechst 33342 were added at a final concentration of 1 mg/mL and cell suspensions were incubated with rotation at room temperature for 30 minutes in the dark. Cells were collected by centrifugation (5,000 X G, 5 min, RT) and washed three times in TBS.

For fluorescence microscopy cells were resuspended in 20 mL solid aqueous mounting medium, mounted on standard microscope slides using coverslips sealed with nail polish. Slides were kept at 4° C in the dark prior to imaging using a model Zeiss X51 microscope equipped with an Infinity 3 camera and INFINITY capture software (Lumenera Corporation). For each sample, five randomly selected fields were imaged in the blue, green, and red channels using uniform exposure times that maximized pixel dynamic range. For image analysis, raw images were imported into ImageJ, versions 1.53k and 2.14.0/1.54f, where two hundred cells were selected randomly using the blue (DNA) channel, then analyzed by counting the number of visible bud scars in the red (WGA-AF555) channel and measuring the cell’s total pixel intensity in the green (EGFP) channel. Data presented is the average of three biological replicate experiments.

For live imaging of cells expressing endogenously tagged *SUR4-mCherry*, cells were grown overnight in Ura- media (to select for LMNA and Progerin expressing plasmids) supplemented with 2% Raf [53]. The next day, the cells were diluted to an OD_600_ of 0.2 in fresh Ura- media supplemented with 2% galactose and incubated at 30° C for 6 hours. A DeltaVision Elite imaging system (GE Healthcare Life Sciences) was used that carried a 60x/1.42 NA oil, Plan Apo N objective (Olympus). Images were collected as 0.2 mm z-stacks using SoftWoRx software (version 6.5.2, GE Healthcare Life Sciences), then rendered and analyzed using Image J (National Institutes of Health). Images were treated using Image J, first by employing the Smooth function, then by using the Unsharp Mask filter (Radius (Sigma): 2.0 pixels, Mask Weight: 0.7-0.8).

Cells for flow cytometry were resuspended in 200 μL TBS following washing as above and stored at 4° C in the dark until analysis. Cells were analyzed on a FACScaliber cytometer. Data analysis, statistics, and figures were generated using the CytExpert software package.

### Statistical methods

Stats packages available in PRISM were used. One-or two-way ANOVA tests were used to compare the means of unmatched groups. Multiple comparisons were performed post hoc using Tukey, Dunnett, or Šídák tests, as indicated, to calculate p-values and assess significance between strains or treatments. Alternatively, an unpaired t-test with Welch's correction was used. Significance was reached where p < 0.01**, p < 0.001***, and p < 0.0001****. When significance was not reached, it was denoted with ns. Values represent ± standard error of the mean (SEM), unless otherwise stated.

## Supplementary Material

Supplementary Figures

## References

[1] Rosswag de Souza S, Böke E, Zaffagnini G. Proteostasis in cellular dormancy: lessons from yeast to oocytes. Trends Biochem Sci. 2025; 50:646–62. 10.1016/j.tibs.2025.05.004 40506311

[2] Hetz C, Dillin A. Central role of the ER proteostasis network in healthy aging. Trends Cell Biol. 2025; 35:548–61. 10.1016/j.tcb.2024.10.003 39547881

[3] Jaunbocus N, Ebenki V, Su H, Slavoff SA. Eukaryotic Microproteins. Annu Rev Biochem. 2025; 94:1–28. 10.1146/annurev-biochem-080124-012840 40245354 PMC12207985

[4] Selvam K, Wyrick JJ, Parra MA. DNA Repair in Nucleosomes: Insights from Histone Modifications and Mutants. Int J Mol Sci. 2024; 25:4393. 10.3390/ijms25084393 38673978 PMC11050016

[5] Barbero-Úriz Ó, Valenti M, Molina M, Fernández-Acero T, Cid VJ. Modeling Necroptotic and Pyroptotic Signaling in Saccharomyces cerevisiae. Biomolecules. 2025; 15:530. 10.3390/biom15040530 40305268 PMC12025182

[6] Godoy P, Hao N. Design principles of gene circuits for longevity. Trends Cell Biol. 2025; 35:840–53. 10.1016/j.tcb.2025.02.006 40082090 PMC12435779

[7] Obata F, Miura M. Regulatory Mechanisms of Aging Through the Nutritional and Metabolic Control of Amino Acid Signaling in Model Organisms. Annu Rev Genet. 2024; 58:19–41. 10.1146/annurev-genet-111523-102042 38857535

[8] Mirisola MG, Longo VD. Yeast Chronological Lifespan: Longevity Regulatory Genes and Mechanisms. Cells. 2022; 11:1714. 10.3390/cells11101714 35626750 PMC9139625

[9] Legon L, Rallis C. Genome-wide screens in yeast models towards understanding chronological lifespan regulation. Brief Funct Genomics. 2022; 21:4–12. 10.1093/bfgp/elab011 33728458 PMC8834652

[10] Shabestary K, Dechant R, Gottschling DE. Along the Trajectory to Understanding Cellular Aging: More Lessons from Yeast. Annu Rev Cell Dev Biol. 2025; 41:353–73. 10.1146/annurev-cellbio-112122-023631 40669496

[11] Lin WH, Damenikan A, Barral Y. The Yin and Yang of replicative aging and rejuvenation. Curr Opin Genet Dev. 2025. 10.1016/j.gde.2025.102375 40555138

[12] Stepchenkova EI, Zadorsky SP, Shumega AR, Aksenova AY. Practical Approaches for the Yeast Saccharomyces cerevisiae Genome Modification. Int J Mol Sci. 2023; 24:11960. 10.3390/ijms241511960 37569333 PMC10419131

[13] Ball DA, Jalloh B, Karpova TS. Impact of Saccharomyces cerevisiae on the Field of Single-Molecule Biophysics. Int J Mol Sci. 2022; 23:15895. 10.3390/ijms232415895 36555532 PMC9781480

[14] Shumaker DK, Dechat T, Kohlmaier A, Adam SA, Bozovsky MR, Erdos MR, Eriksson M, Goldman AE, Khuon S, Collins FS, Jenuwein T, Goldman RD. Mutant nuclear lamin A leads to progressive alterations of epigenetic control in premature aging. Proc Natl Acad Sci USA. 2006; 103:8703–8. 10.1073/pnas.0602569103 16738054 PMC1472659

[15] Arancio W, Pizzolanti G, Genovese SI, Pitrone M, Giordano C. Epigenetic involvement in Hutchinson-Gilford progeria syndrome: a mini-review. Gerontology. 2014; 60:197–203. 10.1159/000357206 24603298

[16] Mattout A, Dechat T, Adam SA, Goldman RD, Gruenbaum Y. Nuclear lamins, diseases and aging. Curr Opin Cell Biol. 2006; 18:335–41. 10.1016/j.ceb.2006.03.007 16632339

[17] Dechat T, Shimi T, Adam SA, Rusinol AE, Andres DA, Spielmann HP, Sinensky MS, Goldman RD. Alterations in mitosis and cell cycle progression caused by a mutant lamin A known to accelerate human aging. Proc Natl Acad Sci USA. 2007; 104:4955–60. 10.1073/pnas.0700854104 17360326 PMC1829246

[18] Goldman RD, Gruenbaum Y, Moir RD, Shumaker DK, Spann TP. Nuclear lamins: building blocks of nuclear architecture. Genes Dev. 2002; 16:533–47. 10.1101/gad.960502 11877373

[19] Almendáriz-Palacios C, Gillespie ZE, Janzen M, Martinez V, Bridger JM, Harkness TA, Mousseau DD, Eskiw CH. The Nuclear Lamina: Protein Accumulation and Disease. Biomedicines. 2020; 8:188. 10.3390/biomedicines8070188 32630170 PMC7400325

[20] Almendáriz-Palacios C, Mousseau DD, Eskiw CH, Gillespie ZE. Still Living Better through Chemistry: An Update on Caloric Restriction and Caloric Restriction Mimetics as Tools to Promote Health and Lifespan. Int J Mol Sci. 2020; 21:9220. 10.3390/ijms21239220 33287232 PMC7729921

[21] Cao K, Blair CD, Faddah DA, Kieckhaefer JE, Olive M, Erdos MR, Nabel EG, Collins FS. Progerin and telomere dysfunction collaborate to trigger cellular senescence in normal human fibroblasts. J Clin Invest. 2011; 121:2833–44. 10.1172/JCI43578 21670498 PMC3223819

[22] Reddy S, Comai L. Lamin A, farnesylation and aging. Exp Cell Res. 2012; 318:1–7. 10.1016/j.yexcr.2011.08.009 21871450 PMC4209918

[23] Ashapkin VV, Kutueva LI, Kurchashova SY, Kireev II. Are There Common Mechanisms Between the Hutchinson-Gilford Progeria Syndrome and Natural Aging? Front Genet. 2019. 10.3389/fgene.2019.00455 31156709 PMC6529819

[24] Wang D, Liu S, Xu S. Identification of hub genes, key pathways, and therapeutic agents in Hutchinson-Gilford Progeria syndrome using bioinformatics analysis. Medicine (Baltimore). 2020; 99:e19022. 10.1097/MD.0000000000019022 32049798 PMC7035007

[25] Köhler F, Bormann F, Raddatz G, Gutekunst J, Corless S, Musch T, Lonsdorf AS, Erhardt S, Lyko F, Rodríguez-Paredes M. Epigenetic deregulation of lamina-associated domains in Hutchinson-Gilford progeria syndrome. Genome Med. 2020; 12:46. 10.1186/s13073-020-00749-y 32450911 PMC7249329

[26] Gillespie ZE, Pickering J, Eskiw CH. Better Living through Chemistry: Caloric Restriction (CR) and CR Mimetics Alter Genome Function to Promote Increased Health and Lifespan. Front Genet. 2016. 10.3389/fgene.2016.00142 27588026 PMC4988992

[27] Boettcher B, Barral Y. The cell biology of open and closed mitosis. Nucleus. 2013. 10.4161/nucl.24676 23644379 PMC3720745

[28] Melcer S, Gruenbaum Y, Krohne G. Invertebrate lamins. Exp Cell Res. 2007; 313:2157–66. 10.1016/j.yexcr.2007.03.004 17451683

[29] Liu H, Wang F, Liu B, Kong F, Fang C. Significance of Raffinose Family Oligosaccharides (RFOs) metabolism in plants. Adv Biotechnol (Singap). 2024; 2:13. 10.1007/s44307-024-00022-y 39883346 PMC11740855

[30] Cao K, Capell BC, Erdos MR, Djabali K, Collins FS. A lamin A protein isoform overexpressed in Hutchinson-Gilford progeria syndrome interferes with mitosis in progeria and normal cells. Proc Natl Acad Sci USA. 2007; 104:4949–54. 10.1073/pnas.0611640104 17360355 PMC1821129

[31] Goldman RD, Shumaker DK, Erdos MR, Eriksson M, Goldman AE, Gordon LB, Gruenbaum Y, Khuon S, Mendez M, Varga R, Collins FS. Accumulation of mutant lamin A causes progressive changes in nuclear architecture in Hutchinson-Gilford progeria syndrome. Proc Natl Acad Sci USA. 2004; 101:8963–8. 10.1073/pnas.0402943101 15184648 PMC428455

[32] Guaragnella N, Ždralević M, Lattanzio P, Marzulli D, Pracheil T, Liu Z, Passarella S, Marra E, Giannattasio S. Yeast growth in raffinose results in resistance to acetic-acid induced programmed cell death mostly due to the activation of the mitochondrial retrograde pathway. Biochim Biophys Acta. 2013; 1833:2765–74. 10.1016/j.bbamcr.2013.07.017 23906793

[33] Postnikoff SD, Harkness TA. Replicative and chronological life-span assays. Methods Mol Biol. 2014. 10.1007/978-1-4939-0799-1_17 24841311

[34] Hilton BA, Liu J, Cartwright BM, Liu Y, Breitman M, Wang Y, Jones R, Tang H, Rusinol A, Musich PR, Zou Y. Progerin sequestration of PCNA promotes replication fork collapse and mislocalization of XPA in laminopathy-related progeroid syndromes. FASEB J. 2017; 31:3882–93. 10.1096/fj.201700014R 28515154 PMC5572696

[35] Ugolini S, Bruschi CV. The red/white colony color assay in the yeast Saccharomyces cerevisiae: epistatic growth advantage of white ade8-18, ade2 cells over red ade2 cells. Curr Genet. 1996; 30:485–92. 10.1007/s002940050160 8939809

[36] Malo ME, Postnikoff SD, Arnason TG, Harkness TA. Mitotic degradation of yeast Fkh1 by the Anaphase Promoting Complex is required for normal longevity, genomic stability and stress resistance. Aging (Albany NY). 2016; 8:810–30. 10.18632/aging.100949 27099939 PMC4925830

[37] Wu D, Yates PA, Zhang H, Cao K. Comparing lamin proteins post-translational relative stability using a 2A peptide-based system reveals elevated resistance of progerin to cellular degradation. Nucleus. 2016; 7:585–96. 10.1080/19491034.2016.1260803 27929926 PMC5214577

[38] Simmons RB. Comparison of chitin localization in Saccharomyces cerevisiae, Cryptococcus neoformans, and Malassezia spp. Mycologiacl Research. 1989. 10.1016/S0953-7562(89)80053-X

[39] Ghavidel A, Baxi K, Prusinkiewicz M, Swan C, Belak ZR, Eskiw CH, Carvalho CE, Harkness TA. Rapid Nuclear Exclusion of Hcm1 in Aging Saccharomyces cerevisiae Leads to Vacuolar Alkalization and Replicative Senescence. G3 (Bethesda). 2018; 8:1579–92. 10.1534/g3.118.200161 29519938 PMC5940150

[40] Bell PJ, Deere D, Shen J, Chapman B, Bissinger PH, Attfield PV, Veal DA. A flow cytometric method for rapid selection of novel industrial yeast hybrids. Appl Environ Microbiol. 1998; 64:1669–72. 10.1128/AEM.64.5.1669-1672.1998 9572934 PMC106213

[41] Model MA, Reese JL, Fraizer GC. Measurement of wheat germ agglutinin binding with a fluorescence microscope. Cytometry A. 2009; 75:874–81. 10.1002/cyto.a.20787 19722258

[42] Chaudhari RD, Stenson JD, Overton TW, Thomas CR. Effect of bud scars on the mechanical properties of Saccharomyces cerevisiae cell walls. Chemical Engineering Science. 2012; 84:188–96. 10.1016/j.ces.2012.08.027

[43] Hyttinen JM, Amadio M, Viiri J, Pascale A, Salminen A, Kaarniranta K. Clearance of misfolded and aggregated proteins by aggrephagy and implications for aggregation diseases. Ageing Res Rev. 2014. 10.1016/j.arr.2014.07.002 25062811

[44] Postnikoff SD, Harkness TA. Mechanistic insights into aging, cell-cycle progression, and stress response. Front Physiol. 2012. 10.3389/fphys.2012.00183 22675309 PMC3366476

[45] Higuchi-Sanabria R, Pernice WM, Vevea JD, Alessi Wolken DM, Boldogh IR, Pon LA. Role of asymmetric cell division in lifespan control in Saccharomyces cerevisiae. FEMS Yeast Res. 2014; 14:1133–46. 10.1111/1567-1364.12216 25263578 PMC4270926

[46] Georgatos SD, Maroulakou I, Blobel G. Lamin A, lamin B, and lamin B receptor analogues in yeast. J Cell Biol. 1989; 108:2069–82. 10.1083/jcb.108.6.2069 2544600 PMC2115584

[47] Ye Q, Worman HJ. Protein-protein interactions between human nuclear lamins expressed in yeast. Exp Cell Res. 1995; 219:292–8. 10.1006/excr.1995.1230 7628545

[48] Spear ED, Hsu ET, Nie L, Carpenter EP, Hrycyna CA, Michaelis S. ZMPSTE24 missense mutations that cause progeroid diseases decrease prelamin A cleavage activity and/or protein stability. Dis Model Mech. 2018; 11:dmm033670. 10.1242/dmm.033670 29794150 PMC6078402

[49] Chang SY, Hudon-Miller SE, Yang SH, Jung HJ, Lee JM, Farber E, Subramanian T, Andres DA, Spielmann HP, Hrycyna CA, Young SG, Fong LG. Inhibitors of protein geranylgeranyltransferase-I lead to prelamin A accumulation in cells by inhibiting ZMPSTE24. J Lipid Res. 2012; 53:1176–82. 10.1194/jlr.M026161 22448028 PMC3351824

[50] Musich PR, Zou Y. DNA-damage accumulation and replicative arrest in Hutchinson-Gilford progeria syndrome. Biochem Soc Trans. 2011; 39:1764–9. 10.1042/BST20110687 22103522 PMC4271832

[51] Osorio FG, Varela I, Lara E, Puente XS, Espada J, Santoro R, Freije JM, Fraga MF, López-Otín C. Nuclear envelope alterations generate an aging-like epigenetic pattern in mice deficient in Zmpste24 metalloprotease. Aging Cell. 2010; 9:947–57. 10.1111/j.1474-9726.2010.00621.x 20961378

[52] Thayer NH, Leverich CK, Fitzgibbon MP, Nelson ZW, Henderson KA, Gafken PR, Hsu JJ, Gottschling DE. Identification of long-lived proteins retained in cells undergoing repeated asymmetric divisions. Proc Natl Acad Sci USA. 2014; 111:14019–26. 10.1073/pnas.1416079111 25228775 PMC4191815

[53] Ptak C, Saik NO, Premashankar A, Lapetina DL, Aitchison JD, Montpetit B, Wozniak RW. Phosphorylation-dependent mitotic SUMOylation drives nuclear envelope-chromatin interactions. J Cell Biol. 2021; 220:e202103036. 10.1083/jcb.202103036 34787675 PMC8641411

[54] Malone CJ, Fixsen WD, Horvitz HR, Han M. UNC-84 localizes to the nuclear envelope and is required for nuclear migration and anchoring during C. elegans development. Development. 1999; 126:3171–81. 10.1242/dev.126.14.3171 10375507

[55] Kwolek-Mirek M, Zadrag-Tecza R. Comparison of methods used for assessing the viability and vitality of yeast cells. FEMS Yeast Res. 2014; 14:1068–79. 10.1111/1567-1364.12202 25154541

