# Supplementary material for: Modeling premature aging in yeast via the expression of Progerin
Source: Aging (Albany NY). 2026 Apr 3;18(1):261–81. doi: 10.18632/aging.206367 (PMC13632848; doi:10.18632/aging.206367)
Supplement: Supplementary file 1 [file aging-18-1-206367-s001.pdf]

SUPPLEMENTARY FIGURES

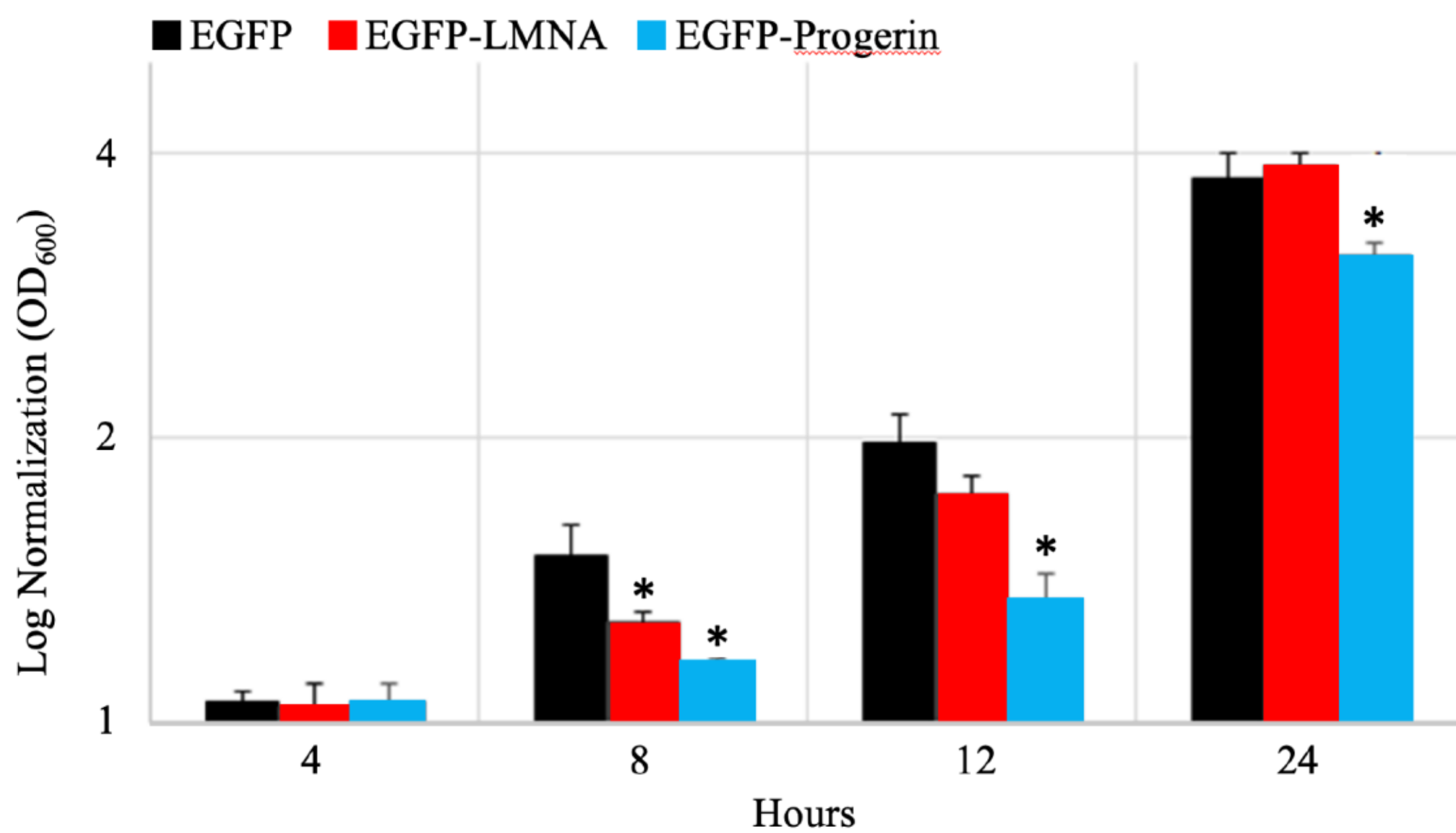

Supplementary Figure 1. Supplemental data linked to Figure 1C. OD_{600} readings for cells after 4, 8, 12 and 24 hours of growth were measured and plotted for cells expressing EGFP (black), EGFP-LMNA (red) or EGFP-Progerin (blue) maintained in media supplemented with 2% Raffinose/0.2% Galactose. SEM is shown. * represents p-values < 0.05 via 2 tailed t-test.

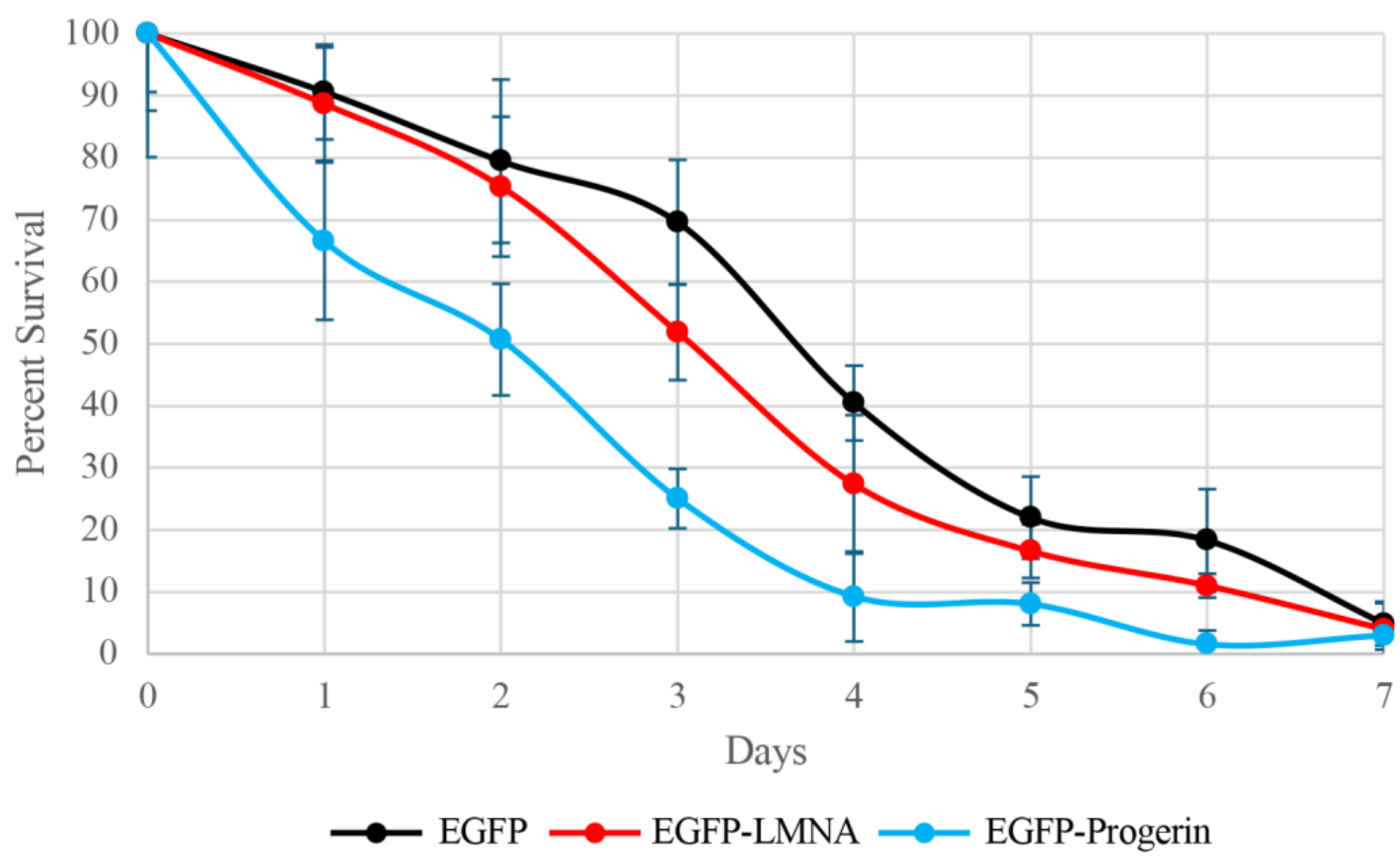

Supplementary Figure 2. Supplemental data linked to Figure 1D. CLS experiment using the transformants shown, grown in 2% RAF/0.2% GAL to stationary phase (Day 0), were performed by removing the same volume of cells every day, which were then diluted and plated onto YPD. Colony forming units were counted, normalized to Day 0, set to 100%, and plotted (n=3). SEM is shown.

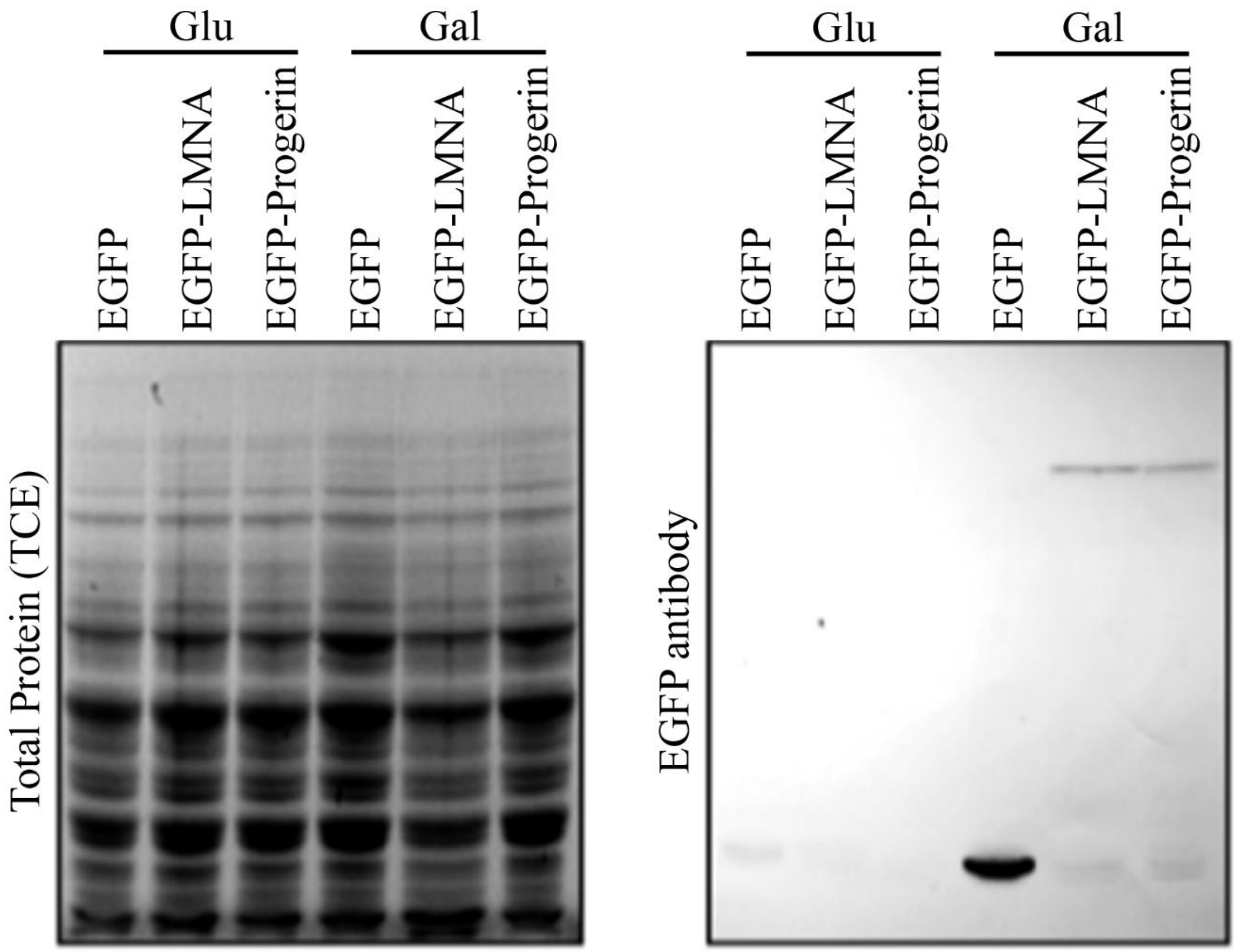

Supplementary Figure 3. Supplementary data linked to Figure 1E. EGFP, EGFP-LMNA or EGFP-Progerin transformed yeast (YPH500) containing the *ade2-101_ochre* point mutation were grown in either glucose containing media (GLU) or induction media (GAL), with proteins extracted. Total protein was evaluated by trichloroethanol (TCE) staining (left panel). Western blots for a-EGFP were also performed (right panel) to verify the expression of EGFP and tagged proteins in this strain. Note that LMNA and Progerin expression, and the number of cells expressing the constructs, varied between the different genetic backgrounds used in this study.
